# The Fib-PNI-MLR Score, an Integrative Model of Coagulation Cascades, Nutrition Status, and Systemic Inflammatory Response, Predicts Urological Outcomes After Surgery in Patients With Non-Metastatic Renal Cell Carcinoma

**DOI:** 10.3389/fonc.2020.555152

**Published:** 2021-01-05

**Authors:** Xiaomin Gao, Yue Pan, Lina Zhou, Yeping Li, Binwei Lin, Yangqin Zheng

**Affiliations:** ^1^ Department of Hematology, The Third Clinical Institute Affiliated to Wenzhou Medical University, People’s Hospital of Wenzhou, Wenzhou, China; ^2^ Department of Urology, Changhai Hospital, Second Military Medical University, Shanghai, China; ^3^ Department of Urology, The First Affiliated Hospital of Wenzhou Medical University, Wenzhou, China; ^4^ Department of Nephrology, The Third Clinical Institute Affiliated to Wenzhou Medical University, People’s Hospital of Wenzhou, Wenzhou, China; ^5^ Department of Urology, Rui’an People’s Hospital, The Third Affiliated Hospital of the Wenzhou Medical University, Wenzhou, China

**Keywords:** inflammation, fibrinogen, nutrition, survival, tumor

## Abstract

Cancer-associated inflammation, activation of coagulation cascades, and malnutrition are closely related to the prognosis of patients with malignancy, including renal cell carcinoma (RCC). This study aimed to investigate the prognostic value of a combination of preoperative plasma fibrinogen, prognostic nutritional index, and monocyte-to-lymphocyte ratio (Fib-PNI-MLR) in patients with non-metastatic RCC undergoing nephrectomy. We retrospectively collected medical data from 829 of the 1,019 cases of RCC. The optimal cutoff values of fibrinogen (≥3.54 *vs.* <3.54, mg/dl), PNI (<47.03 *vs.* ≥47.03), and MLR (≥0.29 *vs.* <0.29) were defined using receiver operating characteristic (ROC) analysis and the Fib-PNI-MLR score (range, 0–3) was determined as the sum of points (0 or 1) assigned to each indicator. As a result, Fib-PNI-MLR was an independent risk factor for overall survival (OS), cancer-specific survival (CSS), and metastatic-free survival (MFS) (all *P* < 0.05). The concordance-index and area under the curve (AUC) were larger for the Fib-PNI-MLR score than that for other clinical parameters. Subgroup analysis (Fuhrman grade G1+G2 and Fuhrman grade G3+G4; pathologic T1, T2, and T3–4 stage) revealed the significant association of a higher Fib-PNI-MLR score with poor urological outcomes (all *P* < 0.05). Data indicated that patients with higher Fib-PNI-MLR might benefit from partial nephrectomy. The Fib-PNI-MLR score might serve as a promising prognostic factor in patients with non-metastatic RCC.

## Introduction

Various traditional prediction factors, including pathologic T stage, Fuhrman grade, and distant metastasis, have been closely associated with patient survival in renal cell carcinoma (RCC) following surgery. Inflammation is vital throughout tumorigenesis, including tumor initiation, promotion, and metastasis (1). The prognostic roles of inflammation-based biomarkers, including neutrophil-to-lymphocyte ratio (NLR), platelet-to-lymphocyte ratio (PLR), and monocyte-to-lymphocyte ratio (MLR), have been confirmed in numerous studies (2–5). These factors are cost-effective and broadly available, and are can be obtained by routine blood testing in many institutions. Preoperative nutritional status is also closely related to the postoperative survival outcomes in patients with RCC (6). Morgan et al. reported that nutrition deficiency, defined as body mass index <18.5 kg/m^2^, albumin <3.5 g/dl, or preoperative weight loss ≥5% of body weight, is associated with poor survival in patients undergoing surgery for RCC (7). Several studies have reported that the preoperative prognostic nutrition index (PNI), a newly proposed tool for assessing the preoperative nutritional status, can be used as an independent prognostic factor to predict survival after nephrectomy in RCC patients (6, 8, 9).

Growing evidence suggests the presence of a relationship between coagulation cascades and tumor biology (10). Fibrinogen, produced by liver, is a key factor involved in coagulation cascades. Elevated preoperative plasma fibrinogen levels can significantly predict tumor metastasis and mortality in patients with RCC following surgery (11, 12). The combined use of biomarkers of inflammation, nutrition status, and coagulation cascades, for prognosis of patients with non-metastatic RCC has not been reported. Presently we aimed to evaluate the clinical significance of a newly established prognostic system featuring, a combination of preoperative plasma fibrinogen (Fib), PNI, and MLR, termed Fib-PNI-MLR, in non-metastatic RCC patients undergoing curative nephrectomy. We also evaluated the association between four groups stratified based on the Fib-PNI-MLR score and the clinical laboratory parameters or clinical pathologic characteristics.

## Materials And Methods

### Population Selection and Data Collection

**Figure 1:**
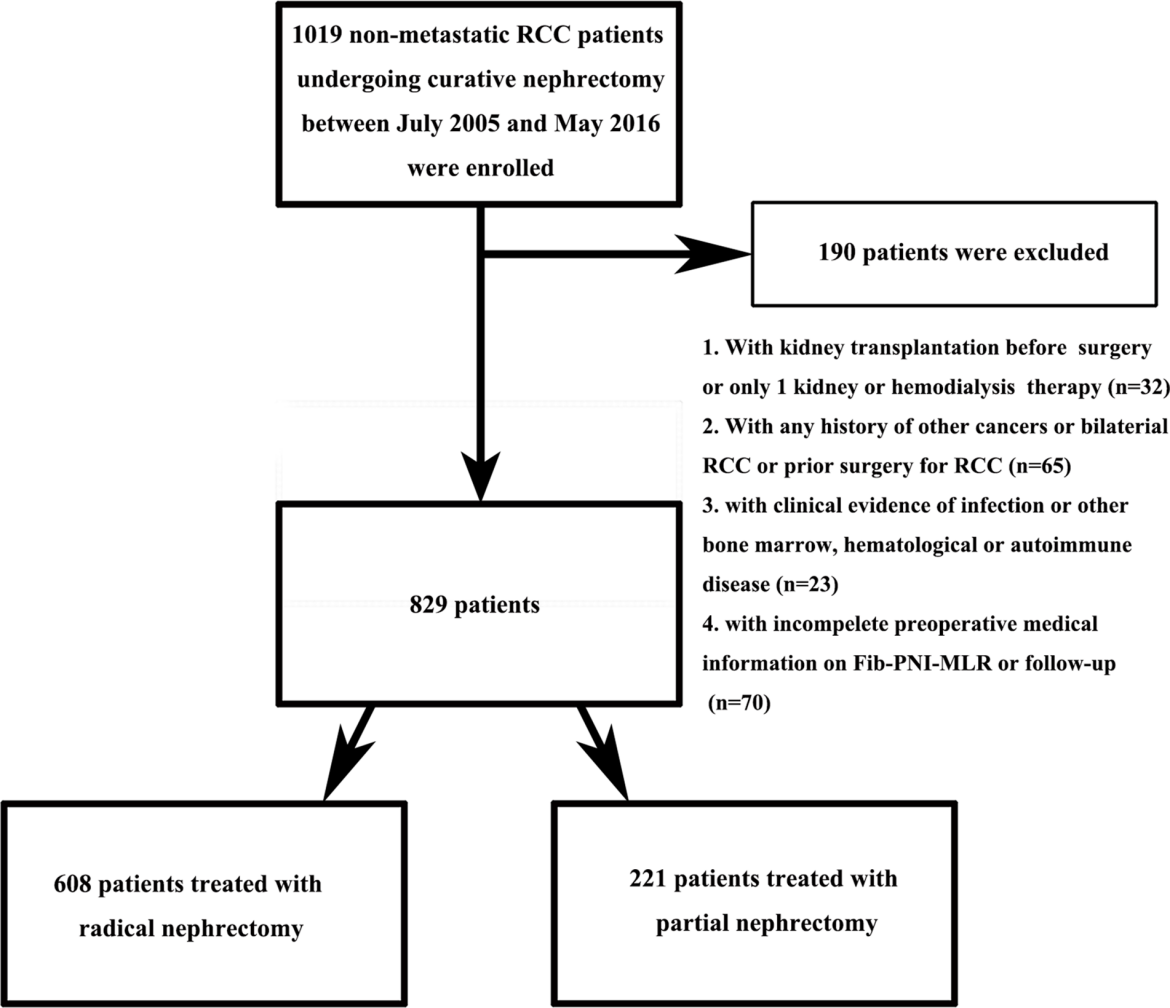
Flowchart showing patient selection.

Between July 2005 and May 2016, a total of 1,019 non-metastatic RCC patients who underwent radical or partial nephrectomy without neo-adjuvant chemotherapy preoperatively at the Urologic Department of our institution were retrospectively reviewed. **Figure 1** showed the inclusion and exclusion criteria to select eligible patients for this study. Patients’ characteristics, including demographics, tumor staging (based on American Joint Committee on Cancer TNM staging, 7^th^ edition), Fuhrman grade, and laboratory assessments, were collected and analyzed. PNI was defined as albumin+5×lymphocyte, NLR as neutrophil/lymphocyte, PLR as platelet/lymphocyte, and MLR as monocyte/lymphocyte. The diabetes mellitus (DM) was defined as the presence of a fasting plasma glucose level of ≥7.0 mmol/L on at least two occasions, a 2-h plasma glucose of ≥11.1 mmol/L in a 75 g oral glucose tolerance test or the requirement for oral hypoglycemic agents and/or insulin to control glucose levels. The hypertension was defined as a systolic blood pressure ≥140 mmHg or a diastolic blood pressure ≥90 mmHg or both and those taking antihypertensive drug. Anemia was defined as serum hemoglobin ≤130 g/dl in man and ≤120 g/dl in woman, hypoalbuminemia as serum albumin <35g/L. Patients were followed up every 3 to 6 months after surgery during the first 2 years for blood and urine testing, cystoscopy, and computed tomography scan or magnetic resonance imaging. Subsequently, patients were advised to visit the doctor annually. Overall survival (OS), cancer-specific survival (CSS), and metastatic-free survival (MFS) were defined as the duration from nephrectomy to mortality due to any-cause, to cancer-specific death, and to the last follow-up of radiologically or histologically confirmed distant metastasis, respectively. Survival was determined based on telephonic interviews, outpatient medical records, or patients’ social security death index. The follow-up ended in September 2016.

### Statistical Analyses

The cutoff values of fibrinogen, PNI, NLR, PLR, and MLR for survival outcomes were determined by receiver operating characteristic (ROC) curve analysis based on the Youden Index. As a result, the optimal cutoff values of fibrinogen, PNI, NLR, PLR, and MLR were 3.54 mg/dl, 47.03, 3.30, 184.16, and 0.29, respectively (**Figures S1** and **S2**). The AUC values of fibrinogen, PNI, NLR, PLR, and MLR were 0.738 (0.669–0.806), 0.735 (0.663–0.808), 0.652 (0.576–0.729), 0.601 (0.519–0.683), and 0.703 (0.628–0.779), respectively. In particular, fibrinogen, PNI, and MLR had higher AUC values than NLR and PLR. Kaplan-Meier analysis was performed to identify the prognostic ability of fibrinogen, PNI, and MLR for urological outcomes after surgery. As shown in **Figures S1** and **S3**, the Kaplan-Meier curves showed significantly worse OS, CSS, and MFS rates in patients with higher fibrinogen (≥3.54 *vs.* <3.54 mg/dl), lower PNI (<47.03 *vs.* ≥47.03), and higher MLR (≥0.29 *vs.* <0.29), suggesting the predictive ability of these factors in RCC patients with respect to survival. To develop the Fib-PNI-MLR score system, each indicator was assigned a score of 0 or 1 based on the cutoff values. The Fib-PNI-MLR score was then calculated as the sum of each factor’s score, which divided the patients into four groups based on scores of 0, 1, 2, or 3 (**Figure S1**).

The comparisons of continuous variables were analyzed using the t-test (normally distributed continuous variables) or Mann-Whitney U test (non-normally distributed data) or one-way ANOVA with an appropriate *post hoc* test while categorical variables were analyzed using Pearson’s chi-square test or Fisher’s exact test. Trends were analyzed using the Cochran-Armitage test. The correlations between fibrinogen and PNI, fibrinogen and MLR, or PNI and MLR were evaluated using Spearman’s rank analysis. Univariate and multivariate analyses were performed to evaluate the hazard ratios (HRs) of significant risk predictors with respect to OS, CSS, and MFS. The discriminative ability of predictive models was assessed using Harrell’s concordance (c)-index and area under the ROC curve (AUC) values. All statistical analyses were performed using SPSS software Version 25.0 (IBM, Armonk, NY, USA). All tests were two-sided, with *P* < 0.05 considered significant.

## Results

### Baseline Characteristics

Ultimately, 829 suitable RCC patients were identified. The baseline characteristics of the patients are summarized in **Tables S1**, **S2**. The study included 526 (63.4%) men and 303 (36.6%) women, with a mean age at surgery of 60.37 ± 12.45 years. The age distribution followed the following pattern: 352 (42.5%) patients ≥65 years and 477 (57.5%) patients <65 years. Partial nephrectomy was performed in 221 (26.7%) cases and radical nephrectomy was performed in 608 (73.3%) cases. A total of 662 (79.9%), 90 (10.8%), 68 (8.2%), and 9 (1.1%) patients presented with pathologic stages T1, T2, T3, and T4, respectively. Furthermore, 281 (33.9%), 358 (43.2%), 170 (20.5%), and 20 (2.4%) patients presented with Fuhrman grade 1, 2, 3, and 4, respectively. The median follow-up duration was 48.8 (range, 32.65–69.30) months. A total of 62 (7.5%) patients died from any cause, 40 (4.8%) patients died from cancer-specific causes, and 82 (9.9%) patients developed metastasis after surgery. The 5-year OS, CSS, and MFS rates were 91.3, 93.9, and 89.8%, respectively.

### Patient Characteristics and Clinical Outcomes Based on the Fibrinogen, Prognostic Nutritional Index, and Monocyte-To-Lymphocyte Ratio Score

**Figure 2:**
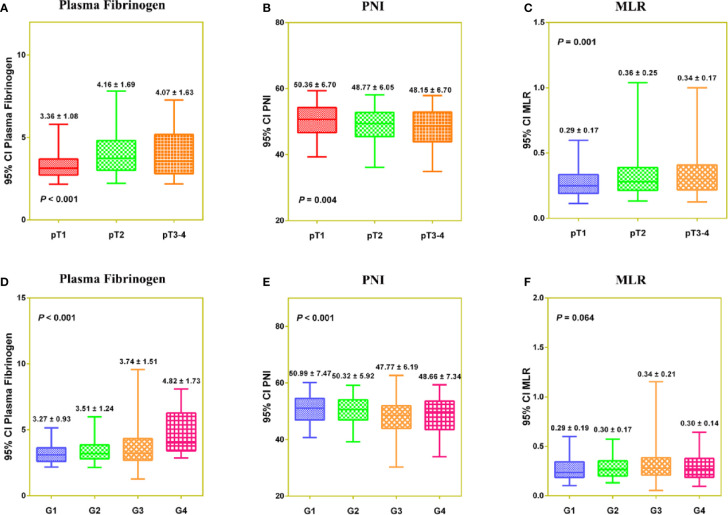
The distribution of Fibrinogen **(A**, **D)**, PNI **(B**, **E)**, and MLR **(C**, **F)** according to pathologic T stage and tumor grade, respectively.

PNI was negatively correlated with fibrinogen (r = −0.273, *P* < 0.001) and MLR (r = −0.469, *P *< 0.001), and fibrinogen was positively correlated with MLR (r = 0.392, *P* < 0.001) (**Figure S4**). In a Venn diagram, Fib-PNI-MLR scores were 0 for 314 (37.9%) patients, 1 for 258 (31.1%) patients, 2 for 162 (19.5%) patients, 3 for 95 (11.5%) patients (**Figure S4D**). As expected, the mean values of Fib-PNI-MLR, fibrinogen, and MLR were higher in patients with higher pathologic T stage and tumor grade, whereas the mean values of PNI were significantly decreased (**Figure S5** and **Figure 2**).

**Table 1** presents data of the association between the clinicopathological parameters or clinical laboratory variables and Fib-PNI-MLR. Significant correlations were evident between Fib-PNI-MLR and age, ASA grade, body mass index (BMI), hypertension, anemia, hypoalbuminemia, surgical approach, chronic kidney disease (CKD) stage, pathologic T stage, Fuhrman grade, tumor necrosis, tumor size, all-cause death, cancer-specific death, metastasis after surgery, 5-year OS, 5-year CSS, 5-year MFS, and follow-up duration of patients who developed metastasis postoperatively (all *P* < 0.05). Significant differences with respect to clinical laboratory parameters among the four Fin-PNI-MLR score groups involved: serum creatinine, blood urea nitrogen (BUN), alkaline phosphatase (ALP), plasma fibrinogen, white blood cells (WBCs), neutrophils, monocytes, lymphocytes, platelets, PNI, NLR, MLR, PLR, hemoglobin, and albumin (all *P* < 0.05).

**Table 1:** Baseline characteristics of patients with non-metastatic RCC according to Fib-PNI-MLR score.

Parameter	Fib-PNI-MLR				
	0 (n = 314)	1 (n = 258)	2 (n = 162)	3 (n = 95)	*P* value
Age, years (≥65/<65)	93/221	111/147	88/74	60/35	<0.001*
Age, years	57.18 ± 11.44	60.00 ± 12.08	64.29 ± 12.61	65.25 ± 13.19	<0.001*
Gender (male/female)	185/129	169/89	105/57	67/28	0.143
ASA grade (1/2/3)	57/249/8	29/210/19	15/134/13	6/73/16	<0.001*
BMI, kg/m^2^ (≥25/<25)	85/229	84/174	35/127	18/77	0.022*
BMI, kg/m^2^	23.38 ± 3.19	23.74 ± 3.31	22.98 ± 3.07	22.54 ± 2.79	0.007*
DM (yes/no)	117/196	99/159	73/89	31/64	0.214
Hypertension (yes/no)	110/204	123/135	76/86	46/49	0.006*
Anemia (yes/no)	8/306	23/235	32/130	41/54	<0.001*
Hypoalbuminemia (yes/no)	2/312	18/240	35/127	41/54	<0.001*
Surgical approach (Partial nephrectomy/Radical nephrectomy)	90/224	88/170	33/129	10/85	<0.001*
CKD stage (CKD1/CKD2/CKD3/CKD4/CKD5)	248/64/2/0/0	182/68/6/0/2	94/54/9/2/3	43/33/11/1/7	<0.001*
Pathologic T stage (pT1/pT2/pT3/pT4)	274/23/15/2	205/28/23/2	125/21/15/1	58/18/15/4	<0.001*
Fuhrman grade (1/2/3/4)	121/140/48/5	89/110/53/6	51/66/42/3	20/42/27/6	0.005*
Histologic subtype (Clear cell/Papillary/Chromophobe/Collecting duct/Unclassified)	271/17/25/0/1	226/15/16/0/1	136/16/9/0/1	81/9/4/1/0	0.576
Tumor necrosis (yes/no)	7/307	8/250	8/154	9/86	0.011*
Tumor size, cm (≥7/<7)	33/281	41/217	34/128	33/62	<0.001*
Tumor size, cm	4.34 ± 2.41	4.60 ± 2.78	5.21 ± 4.26	6.07 ± 4.01	<0.001*
Serum creatinine, mg/dl	0.75 ± 0.16	0.83 ± 0.61	1.00 ± 1.25	1.61 ± 2.50	<0.001*
BUN, mg/dl	5.40 ± 1.33	5.86 ± 2.19	6.24 ± 3.52	6.83 ± 4.82	<0.001*
Uric acid, mg/dl	5.65 ± 1.45	5.61 ± 1.52	5.56 ± 1.57	5.74 ± 1.65	0.793
ALT, U/L	25.09 ± 18.92	25.85 ± 22.84	27.31 ± 31.40	25.16 ± 35.70	0.827
AST, U/L	24.62 ± 11.85	25.93 ± 15.09	28.13 ± 18.88	25.98 ± 18.49	0.130
ALP, U/L	76.59 ± 25.65	78.10 ± 25.10	80.78 ± 32.07	96.34 ± 71.48	<0.001*
Plasma fibrinogen (≥3.54/<3.54), mg/dl	0/314	87/171	104/58	95/0	<0.001*
WBCs, cells × 10^3^/ul	6.38 ± 1.79	6.36 ± 1.77	6.59 ± 2.37	7.66 ± 2.66	<0.001*
Neutrophils, cells/ul	3.68 ± 1.23	3.96 ± 1.37	4.41 ± 2.19	5.30 ± 2.12	<0.001*
Monocytes, cells/ul	0.40 ± 0.13	0.48 ± 0.18	0.55 ± 0.21	0.68 ± 0.28	<0.001*
Lymphocytes, cells/ul	2.14 ± 0.99	1.77 ± 0.52	1.47 ± 0.49	1.30 ± 0.38	<0.001*
Platelets, cells × 10^4^/ul	218.18 ± 60.05	215.69 ± 68.73	218.10 ± 81.63	248.75 ± 83.07	0.001*
PNI (<47.03/≥47.03)	0/314	58/200	85/77	95/0	<0.001*
NLR (≥3.30/<3.30)	15/299	36/222	52/110	54/41	<0.001*
MLR (≥0.29/<0.29)	0/314	113/145	135/27	95/0	<0.001*
PLR (≥184.16/<184.16)	18/296	32/226	46/116	51/44	<0.001*
Hemoglobin, g/dl	138.22 ± 14.53	135.81 ± 16.72	129.94 ± 17.60	118.11 ± 23.88	<0.001*
Albumin, g/L	43.23 ± 3.56	41.70 ± 4.18	39.01 ± 5.63	35.07 ± 3.88	<0.001*
All-cause death, n (%)	6/308	8/250	19/143	29/66	<0.001*
Follow-up duration, months, median (quartile)	55.06 ± 27.69	55.27 ± 27.46	52.13 ± 28.23	49.93 ± 29.69	0.294
5-year OS rate	97.5%	97.1%	86.3%	66.2%	<0.001*
Cancer-specific death, n	3/311	4/254	13/149	20/75	<0.001*
Follow-up duration, months, median (quartile)	55.06 ± 27.69	55.27 ± 27.46	52.13 ± 28.23	49.93 ± 29.69	0.294
5-year CSS rate	98.7%	98.1%	89.9%	74.1%	<0.001*
Patients who developed metastasis after surgery, n	17/297	15/243	18/144	32/63	<0.001*
Follow-up duration, months, median (quartile)	54.03 ± 27.57	53.61 ± 26.95	50.57 ± 29.19	43.90 ± 31.93	0.014*
5-year MFS rate	95.2%	94.3%	89.0%	61.5%	<0.001*

*P < 0.05.

RCC, renal cell carcinoma; DM, diabetes mellitus; CKD, chronic kidney disease; OS, overall survival; CSS, cancer-specific survival; MFS, metastatic-free survival; WBC, white blood cell; PNI, prognostic nutritional index; NLR, neutrophil-lymphocyte ratio; PLR, platelet-lymphocyte ratio; MLR, monocyte-lymphocyte ratio; ALT, alanine aminotransferase; AST, glutamate aminotransferase; ALP, alkaline phosphatase; BUN, urea nitrogen.

### Survival and Cox Regression Analysis of Fibrinogen, Prognostic Nutritional Index, and Monocyte-To-Lymphocyte Ratio for Overall Survival, Cancer-Specific Survival, and Metastatic-Free Survival

**Figure 3:**
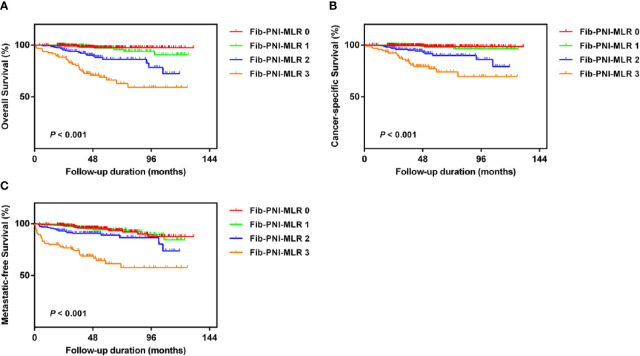
Kaplan-Meier analysis for overall survival (OS) **(A)**, cancer-specific survival (CSS) **(B)**, and metastatic-free survival (MFS) **(C)** in RCC patients based on Fib-PNI-MLR score (low-risk: Fib-PNI-MLR score = 0 or 1; moderate risk: Fib-PNI-MLR score = 2; high risk: Fib-PNI-MLR score = 3).

**Figure 4:**
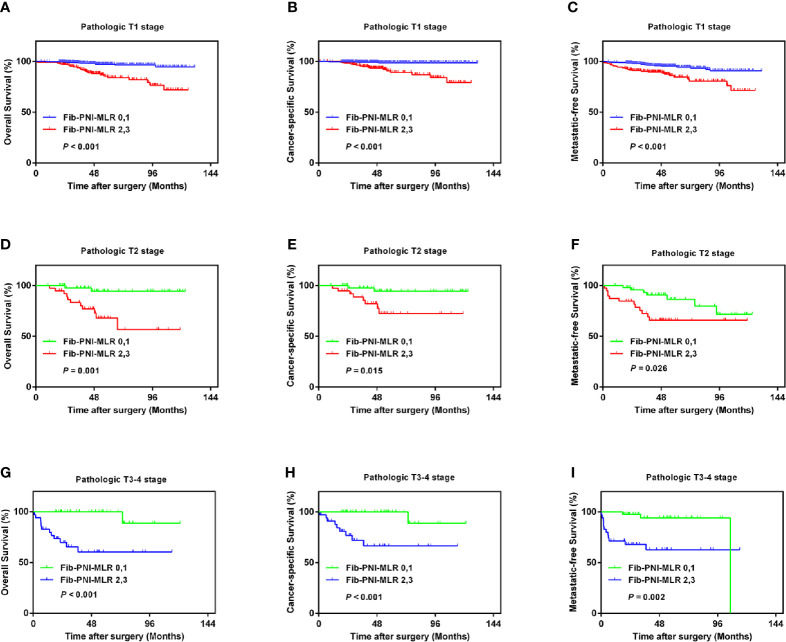
Kaplan-Meier analysis shows that the OS, CSS, and MFS of patients with Fib-PNI-MLR scores of 2 or 3 were lower than those with Fib-PNI-MLR scores of 0 or 1 under adjusted pathologic T stage [T1 **(A–C)**, T2 **(D–F)**, and T3-4 **(G–I)**].

**Figure 5:**
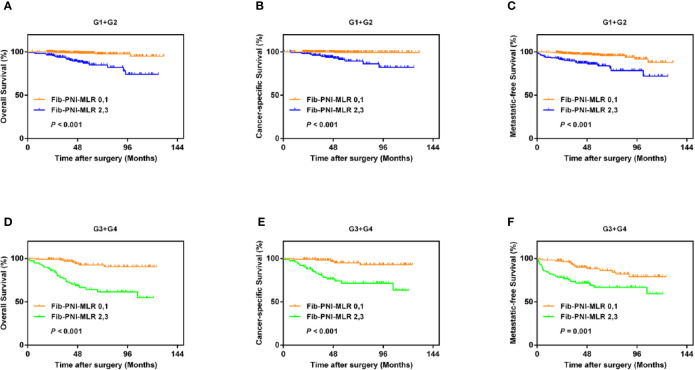
Kaplan-Meier analysis shows that the OS, CSS, and MFS of patients with Fib-PNI-MLR scores of 2 or 3 were lower than those with Fib-PNI-MLR scores of 0 or 1 under tumor grade [G1+G2 **(A–C)** and G3+G4 **(D–F)**].

Kaplan-Meier analysis and log-rank tests showed that patients with an Fib-PNI-MLR score of 3 displayed significantly shorter 5-year OS, CSS, and MFS compared with patients with scores of 2, 1, or 0 (OS: 66.2 *vs.* 86.3 *vs.* 97.1 *vs.* 97.5%, *P* < 0.001; CSS: 74.1 *vs.* 89.9 *vs.* 98.1 *vs.* 98.7%, *P* < 0.001; MFS: 61.5 *vs.* 89.0 *vs.* 94.3 *vs.* 95.2%, respectively; *P* < 0.001) (**Figure 3**). Moreover, patients can be stratified into low-risk (score 0 or 1), moderate-risk (score 2), and high-risk (score 3) group indicated by **Figure 3**. In the subgroup analysis stratified based on pathologic T stage (pT1, pT2, and pT3–4), the OS, CSS, and MFS of patients with Fib-PNI-MLR scores of 2 or 3 were lower than those with Fib-PNI-MLR scores of 0 or 1 (pT1 stage: *P* < 0.001 for OS, CSS, and MFS; pT2 stage: *P* = 0.001 for OS, *P* = 0.015 for CSS, and *P* = 0.026 for MFS; pT3–4 stage: *P* < 0.001 for OS and CSS, *P* = 0.002 for MFS) (**Figure 4**). Further analyses were performed in subgroups based on tumor grade (G1+G2 and G3+G4). Patients with a Fib-PNI-MLR score of 2 or 3 had worse OS, CSS, and MFS rates than those with a score of 0 or 1 in groups G1+G2 (*P* < 0.001 for OS, CSS, and MFS) and G3+G4 (*P* < 0.001 for OS and CSS, *P* = 0.001 for MFS) (**Figure 5**). In univariate and multivariate analyses, BMI, pT stage, and tumor grade were significantly associated with OS, CSS, and MFS (all *P* < 0.05) (**Table S3** and **Table 2**). As expected, the Fib-PNI-MLR score was an independent risk predictor of OS (HR = 6.471, 95% CI, 2.969–14.107, *P* < 0.001), CSS (HR = 9.807, 95% CI, 3.622–26.548, *P* < 0.001), and MFS (HR = 3.368, 95% CI, 1.781–6.371, *P* < 0.001).

**Table 2:** Multivariate analysis of parameters for the prediction of survival outcomes in 829 non-metastatic RCC patients.

Parameter	Overall survival			Cancer-specific survival			Metastasis-free survival		
	HR	95% CI	*P* value	HR	95% CI	*P* value	HR	95% CI	*P* value
Age, years (≥65/<65)	1.746	0.963–3.166	0.066	1.484	0.725–3.037	0.280	1.379	0.849–2.239	0.194
Gender (male/female)		–			–		1.487	0.878–2.517	0.140
ASA grade (≥3/<3)	1.861	0.930–3.724	0.079	2.254	0.897–5.663	0.084	1.199	0.605–2.376	0.604
BMI, kg/m^2^ (≥25/<25)	0.504	0.217–1.168	0.110		–		0.403	0.202–0.803	0.010*
Anemia (yes/no)	1.421	0.777–2.597	0.254	0.976	0.460–2.071	0.949	0.970	0.538–1.751	0.920
Hypoalbuminemia (yes/no)	0.973	0.535–1.772	0.930	0.697	0.317–1.529	0.368	0.789	0.437–1.424	0.432
Surgical approach (Partial nephrectomy/Radical nephrectomy)		–		0.484	0.135–1.744	0.267		–	
CKD stage									
CKD1	1.000	Reference	1.000	1.000	Reference	1.000	1.000	Reference	1.000
CKD2–3 *vs.* CKD1	1.541	0.556–4.270	0.406	1.897	0.602–5.978	0.274	1.251	0.470–3.330	0.654
CKD4–5 *vs.* CKD1	2.611	0.775–8.795	0.122	0.930	0.114–7.596	0.946	2.579	0.902–7.370	0.077
Pathologic T stage									
pT1	1.000	Reference	1.000	1.000	Reference	1.000	1.000	Reference	1.000
pT2 *vs* pT1	1.454	0.591–3.571	0.415	1.279	0.445–3.674	0.648	1.770	0.798–3.926	0.160
pT3 *vs* pT1	2.340	1.066–5.136	0.034*	2.554	0.982–6.641	0.055	2.234	1.122–4.451	0.022*
pT4 *vs* pT1	8.658	2.253–33.274	0.002*	8.127	1.568–42.112	0.013*	6.379	1.777–22.895	0.004*
Fuhrman grade (≥3/<3)	2.386	1.364–4.173	0.002*	3.106	1.532–6.296	0.002*	1.891	1.172–3.052	0.009*
Tumor necrosis (yes/no)		–		0.940	0.285–3.097	0.918		–	
Tumor size, cm (≥7/<7)	1.040	0.479–2.257	0.921	1.497	0.600–3.737	0.387	1.030	0.517–2.051	0.933
Fib-PNI-MLR									
0–1	1.000	Reference	1.000	1.000	Reference	1.000	1.000	Reference	1.000
2 *vs.* 0–1	3.588	1.695–7.597	0.001*	5.181	1.911–14.046	0.001*	1.426	0.756–2.691	0.273
3 *vs.* 0–1	6.471	2.969–14.107	<0.001*	9.807	3.622–26.548	<0.001*	3.368	1.781–6.371	<0.001*
NLR (≥3.30/<3.30)	1.274	0.724–2.240	0.401	1.464	0.716–2.992	0.296	1.533	0.911–2.578	0.107
PLR (≥184.16/<184.16)	0.805	0.436–1.487	0.489	0.954	0.463–1.963	0.897	1.223	0.712–2.099	0.466
BUN (≥7.2/<7.2), mg/dl	0.979	0.466–2.058	0.955		–		1.464	0.796–2.693	0.220

*P < 0.05.

RCC, renal cell carcinoma; DM, diabetes mellitus; CKD, chronic kidney disease; OS, overall survival; CSS, cancer-specific survival; MFS, metastatic-free survival; PNI, prognostic nutritional index; NLR, neutrophil-lymphocyte ratio; PLR, platelet-lymphocyte ratio; BUN, urea nitrogen.

### Predictive Ability of Fibrinogen, Prognostic Nutritional Index, and Monocyte-To-Lymphocyte Ratio Compared to That of Other Parameters

Previous studies have suggested that inflammation-based factors can serve as independent predictors of prognosis (2, 13). Therefore, in the present study, we evaluated the predictive value of NLR and PLR without including Fib-PNI-MLR in patients with RCC. Multivariate analysis demonstrated that NLR was significantly associated with OS, CSS, and MFS, but not with PLR (**Table S4**). C-index and ROC curves analyses were performed to compare the clinical implications of these significant independent factors, including NLR, Fib-PNI-MLR, pT stage, and tumor grade. The c-index and AUC values of Fib-PNI-MLR were 0.787 (0.730–0.844) and 0.791 (0.730–0.853) for predicting OS, 0.810 (0.744–0.876) and 0.810 (0.740–0.880) for predicting CSS, and 0.719 (0.660–0.778) and 0.697 (0.630–0.764) for predicting MFS, respectively. These values were higher than those of NLR, pT stage, and tumor grade (**Table 3**). Furthermore, pT stage and tumor grade had higher c-index and AUC values when combined with Fib-PNI-MLR. These data indicated the potential value of Fib-PNI-MLR in clinical settings for the risk stratification of patients.

**Table 3:** C-index and ROC analysis for the prognostic accuracy of Fib-PNI-MLR and other variables for OS, CSS, and MFS.

Variables	c-index(95% CI)	AUC(95% CI)	Sensitivity%	Specificity%	Youden index	Positive likelihood ratio	Negative likelihood ratio
OS							
Fib-PNI-MLR	0.787 (0.730–0.844)	0.791 (0.730–0.853)	77.4%	72.8%	0.502	2.841	0.31
NLR	0.640 (0.573–0.707)	0.652 (0.576–0.729)	41.9%	82.9%	0.248	2.45	0.70
Pathologic T stage	0.658 (0.588–0.728)	0.621 (0.542–0.700)	41.9%	81.6%	0.235	2.28	0.71
Fuhrman grade	0.664 (0.598–0.730)	0.703 (0.637–0.770)	53.2%	79.5%	0.327	2.60	0.59
pT+Fib-PNI-MLR	0.808 (0.747–0.869)	0.803 (0.741–0.865)	82.5%	71.6%	0.541	2.90	0.24
Grade+Fib-PNI-MLR	0.823 (0.770–0.876)	0.835 (0.781–0.888)	74.2%	78.4%	0.526	3.43	0.33
CSS							
Fib-PNI-MLR	0.810 (0.744–0.876)	0.810 (0.740–0.880)	82.5%	71.6%	0.541	2.90	0.24
NLR	0.687 (0.606–0.768)	0.679 (0.584–0.774)	65.0%	69.5%	0.345	2.13	0.50
Pathologic T stage	0.700 (0.616–0.784)	0.660 (0.564–0.756)	50.0%	81.4%	0.314	2.69	0.61
Fuhrman grade	0.700 (0.621–0.779)	0.727 (0.647–0.807)	60.0%	79.0%	0.390	2.86	0.51
pT+Fib-PNI-MLR	0.842 (0.776–0.908)	0.835 (0.770–0.900)	82.5%	71.6%	0.541	2.90	0.24
Grade+Fib-PNI-MLR	0.855 (0.800–0.910)	0.861 (0.804–0.918)	90.0%	70.3%	0.603	3.03	0.14
MFS							
Fib-PNI-MLR	0.719 (0.660–0.778)	0.697 (0.630–0.764)	61.0%	72.3%	0.333	2.20	0.54
NLR	0.635 (0.578–0.692)	0.653 (0.587–0.720)	58.5%	67.2%	0.257	1.78	0.62
BMI	0.576 (0.537–0.611)	0.586 (0.530–0.642)	41.8%	78.0%	0.198	1.90	0.75
CKD stage	0.572 (0.526–0.618)	0.588 (0.519–0.658)	45.1%	69.9%	0.150	1.50	0.79
Pathologic T stage	0.637 (0.578–0.696)	0.618 (0.549–0.687)	41.5%	82.2%	0.237	2.33	0.71
Fuhrman grade	0.630 (0.572–0.688)	0.674 (0.613–0.736)	47.6%	79.8%	0.274	2.36	0.66
pT+Fib-PNI-MLR	0.745 (0.684–0.806)	0.733 (0.669–0.796)	57.3%	81.3%	0.386	3.06	0.53
Grade+Fib-PNI-MLR	0.759 (0.702–0.816)	0.771 (0.714–0.828)	78.0%	64.0%	0.440	2.29	0.34

RCC, renal cell carcinoma; CKD, chronic kidney disease; OS, overall survival; CSS, cancer-specific survival; MFS, metastatic-free survival; PNI, prognostic nutritional index; NLR, neutrophil-lymphocyte ratio.

### Association Between Operative Procedure Options

**Figure 6:**
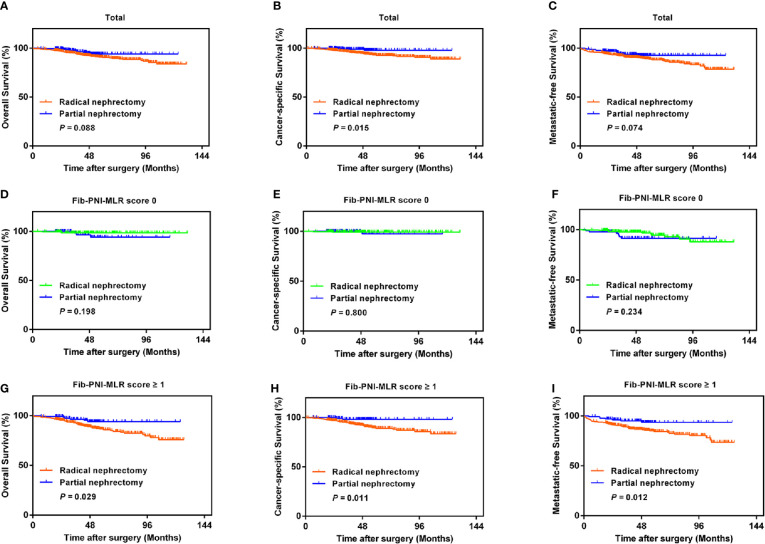
Kaplan-Meier analysis of OS, CSS, and MFS according to the treatment options in patients with all of the Fib-PNI-MLR **(A–C)**, the Fib-PNI-MLR 0 or 1 **(D–F)**, and the Fib-PNI-MLR 2 or 3 **(G–I)**, respectively.

With the improvement in surgical techniques and with an increase in doctor experience, an increasing number of urologists prefer partial nephrectomy for patients with RCC (14). However, cancer control may be undermined following partial nephrectomy, especially in case of large renal tumors, compared to that observed in response to radical nephrectomy (15). We performed Kaplan-Meier analysis and log-rank test to compare the urological outcomes of partial nephrectomy and radical nephrectomy, and further assessed the treatment outcomes in RCC based on the Fib-PNI-MLR score. Patients undergoing partial nephrectomy had significantly favorable CSS (*P* = 0.015) compared with those undergoing radical nephrectomy and had the trend of better OS (*P* = 0.088) and MFS (*P* = 0.074) (**Figures 6A–C**) even though these values were not significant. We then stratified the patients into Fib-PNI-MLR scores of 0, and ≥1 to assess whether the Fib-PNI-MLR score was associated with the treatment outcomes. In the subgroup of patients with Fib-PNI-MLR = 0, the survival outcomes of the two surgical options were comparable with respect to OS (*P* = 0.198), CSS (*P* = 0.800), and MFS (*P* = 0.234) (**Figures 6D–F**). However, patients treated with partial nephrectomy displayed significantly better OS (*P* = 0.029), CSS (*P* = 0.011), and MFS (*P* = 0.012) compared with those undergoing radical nephrectomy (**Figures 6G–I**) in patients with Fib-PNI-MLR ≥ 1. These results indicated that patients with a Fib-PNI-MLR ≥1 who underwent radical nephrectomy were more likely to have a poor prognosis.

## Discussion

We evaluated the prognostic impact of the newly developed, Fib-PNI-MLR assessment tool, in patients with non-metastatic RCC treated with partial or radical nephrectomy. An increased Fib-PNI-MLR score was significantly associated with shorter OS, CSS, and MFS. The findings identified Fib-PNI-MLR was an independent risk factor for patients undergoing nephrectomy.

Accumulating evidence indicates that a systemic inflammatory response in the tumor cells, malnutrition status, and coagulation cascades are important for the tumor growth, angiogenesis, progression, and metastasis and are closely related to the prognosis of cancer patients (1, 6, 10). Lymphocytes, especially CD4+ T cells, play a pivotal role in the immune response to tumor cells. Circulating lymphocytes have anti-tumor effects and improve survival outcomes in cancer patients by secreting several cytokines, such as interferon-gamma (IFN-γ) and tumor necrosis factor-alpha (TNF-α) (16). Relatively low levels of lymphocytes may impair cancer immune surveillance and defense, which results in markedly reduced anti-tumor effects (16, 17). Circulating monocyte-derived macrophages are the most common type of tumor-infiltrating immune cells, which contribute to early carcinogenesis and tumor progression (18). These cells will be educated to become protumoral cells once the tumors are established (19). Macrophages are the major component of the immune cell infiltrate in the tumor microenvironment, constituting up to 50% of the tumor mass (18, 20, 21). Therefore, to some extent, the level of monocytes can reflect the tumor burden in cancer patients (22) and monocyte-derived macrophage density has been correlated with poor prognosis (17). Therefore, RCC patients with high MLR are more likely to experience adverse urological outcomes.

Preoperative malnutrition status is generally considered to influence the survival outcomes in patients with malignant tumors. Patients with nutrition deficiency are vulnerable to infection or cancer *via* cell-mediated mechanisms and other immune pathways, which will delay surgery or adjuvant therapy (23, 24). A previous study prospectively evaluated the risk of malnutrition using the Nutritional Risk Screening (NRS) 2002. The authors reported that 16% of the urological patients were at a severe risk of malnutrition, and malignant disease was a significant factor for a greater risk of malnutrition (25). In the present study, PNI, calculated by lymphocyte count and serum albumin, was used to assess the preoperative nutritional status. PNI was confirmed as a strong independent predictor of survival in RCC patients. In addition, a close relationship between hyperfibrinogenemia and tumor progression has been demonstrated in several studies (10, 11). Fibrinogen is synthesized as an acute-phase protein, which can regulate the systemic inflammatory state and cancer cell progression (26). Furthermore, tumor cells can secrete fibrinogen by producing interleukin-6 to promote the proliferation of fibroblast growth factor-2 (27, 28). Fibrinogen may mediate the adhesion of cancer cells to platelets, followed by the gradual formation of tumor thrombi and binding to endothelial cells. WBCs and pro-inflammatory cytokines can also bind to the endothelium with the help of fibrinogen, thereby inducing tumor aggressiveness (29). Consistent with this evidence, patients with higher fibrinogen levels were correlated with worse OS, CSS, and MFS in this study.

The combination of fibrinogen, PNI, and MLR had favorable predictive value for patients with RCC. The Fib-PNI-MLR score improved the limited effect of fibrinogen, PNI, or MLR alone on the survival of patients after surgery and eventually increased the predictive significance for tumor progression. We additionally performed a subgroup analysis by stratifying patients according to pathologic T stage. The OS, CSS, and MFS in patients with Fib-PNI-MLR scores of 2 or 3 were shorter than those of patients with a score of 0 or 1 in pathologic stage T1, T2, and T3–4. We also found that the Fib-PNI-MLR score was significantly associated with OS, CSS, and MFS in subgroup tumor grades 1–2 and tumor grades 3–4. In addition, this new predictive tool was closely related to pathologic T stage, Fuhrman grade, tumor necrosis, tumor size. Therefore, to better understanding the pathologic T stage and Fuhrman grade status on patient survival and tumor progression, we recommend considering the Fib-PNI-MLR score for RCC patients.

Partial nephrectomy, also known as nephron-sparing surgery, is being increasingly recommended for non-metastatic patients based on prior descriptions of similar oncological control and long-term renal preservation effect compared with that observed on using radical nephrectomy (14, 30, 31). However, there was no improvement in surgical techniques in partial nephrectomy and the many flaws, including low recruitment, selection and verification biases, and different learning curves, in these reports undermined (32). Therefore, efforts should be made to identify patients who might benefit from partial nephrectomy with respect to postoperative survival (33). Consistent with previous findings, patients undergoing partial nephrectomy had better rates of OS, CSS, and MFS than those treated with radical nephrectomy in the present study. However, for patients with a Fib-PNI-MLR score of 0, the survival outcomes were similar for partial nephrectomy and radical nephrectomy. For patients with Fib-PNI-MLR ≥1, partial nephrectomy significantly improved survival. The overall data indicate that Fib-PNI-MLR can be used as a preoperative assessment tool in the clinic for urologists to identify patients who should receive partial nephrectomy.

This study has several limitations. First, it was a retrospective study from a single institute with selective bias. Second, although some undetected confounding factors have been restricted, it is possible that the Fib-PNI-MLR score can be influenced by many parameters. In addition, it was impossible for us to include all potential related factors, such as C-reactive protein and D-dimer, for Cox regression analysis. Third, we did not include patients with metastasis before surgery and the findings cannot be generalized to all RCC patients.

In conclusion, high Fib-PNI-MLR score is a significant independent risk factor for OS, CSS, and MFS in patients with non-metastatic RCC undergoing curative nephrectomy. We hope that economical and reliable assessment tool will help urologists better stratify patients and guide the therapeutic strategies postoperatively.

## Data Availability Statement

The raw data supporting the conclusions of this article will be made available by the authors, without undue reservation, on reasonable request.

## Ethics Statement

The studies involving human participants were reviewed and approved by the ethics committee of The First Affiliated Hospital of Wenzhou Medical University. The patients/participants provided their written informed consent to participate in this study.

## Author Contributions

YZ, BL, and YL conceived and designed the study. XG, YP, and YL obtained the data. XG analyzed, interpreted, and drafted the manuscript. YZ and LZ revised the article. All authors contributed to the article and approved the submitted version. They are also accountable for all aspects of the work.

## Conflict of Interest

The authors declare that the research was conducted in the absence of any commercial or financial relationships that could be construed as a potential conflict of interest.

## Supplementary Material

Supplementary Figure 1 The Fib-PNI-MLR score system was developed based on fibrinogen, PNI, and MLR. The optimal cutoff values of individual component were determined by performing receiver operating characteristic (ROC) curve analysis using the Youden index. Kaplan-Meier curves showed patients’ overall survival (OS) with higher score (score 1) indicating poorer prognosis. The Fib-PNI-MLR score was determined as the sum score of 0 or 1 according to each cutoff value.

Supplementary Figure 2 ROC curves for NLR **(A)** and PLR **(B)**. The optimal cutoff values of NLR and PLR were determined by performing receiver operating characteristic (ROC) curve analysis using the Youden index.

Supplementary Figure 3 Kaplan-Meier analysis of CSS and MFS in patients stratified by fibrinogen, MLR, and PLR.

Supplementary Figure 4 Significant correlation between fibrinogen and PNI **(A)** (r = −0.273, *P* < 0.001), fibrinogen and MLR **(B)** (r = 0.392, *P* < 0.001), PNI and MLR **(C)** (r = −0.469, *P* < 0.001). In addition, a venn diagram of each population according to the Fib-PNI-MLR score was shown **(D).**

Supplementary Figure 5 The distribution of Fib-PNI-MLR score according to tumor grade **(A)** and pathologic T stage **(B)**, respectively.

Supplementary Figure 6 Kaplan-Meier analysis shows that the OS, CSS, and MFS of patients with fibrinogen scores of 1 were lower than those with fibrinogen scores of 0 under adjusted pathologic T stage (T1, T2, and T3–4).

Supplementary Figure 7 Kaplan-Meier analysis shows that the OS, CSS, and MFS of patients with fibrinogen scores of 1 were lower than those with fibrinogen scores of 0 under tumor grade (G1+G2 and G3+G4).

Supplementary Figure 8 Kaplan-Meier analysis of OS, CSS, and MFS according to the treatment options in patients with all of the fibrinogen **(A–C)**, the fibrinogen score 0 **(D–F)**, and the fibrinogen score 1 **(G–I)**, respectively.

Supplementary Figure 9 Kaplan-Meier analysis shows that the OS, CSS, and MFS of patients with PNI scores of 1 were lower than those with PNI scores of 0 under adjusted pathologic T stage (T1, T2, and T3–4).

Supplementary Figure 10 Kaplan-Meier analysis shows that the OS, CSS, and MFS of patients with PNI scores of 1 were lower than those with PNI scores of 0 under tumor grade (G1+G2 and G3+G4).

Supplementary Figure 11 Kaplan-Meier analysis of OS, CSS, and MFS according to the treatment options in patients with all of the PNI **(A–C)**, the PNI score 0 **(D–F)**, and the PNI score 1 **(G–I)**, respectively.

Supplementary Figure 12 Kaplan-Meier analysis shows that the OS, CSS, and MFS of patients with MLR scores of 1 were lower than those with MLR scores of 0 under adjusted pathologic T stage (T1, T2, and T3–4).

Supplementary Figure 13 Kaplan-Meier analysis shows that the OS, CSS, and MFS of patients with MLR scores of 1 were lower than those with MLR scores of 0 under tumor grade (G1+G2 and G3+G4).

Supplementary Figure 14 Kaplan-Meier analysis of OS, CSS, and MFS according to the treatment options in patients with all of the MLR **(A–C)**, the MLR score 0 **(D–F)**, and the MLR score 1 **(G–I)**, respectively.
