# Supplementary figures and images for: The Fib-PNI-MLR Score, an Integrative Model of Coagulation Cascades, Nutrition Status, and Systemic Inflammatory Response, Predicts Urological Outcomes After Surgery in Patients With Non-Metastatic Renal Cell Carcinoma
Source: Front Oncol. 2021 Jan 5;10:555152. doi: 10.3389/fonc.2020.555152 (PMC7819501; doi:10.3389/fonc.2020.555152)

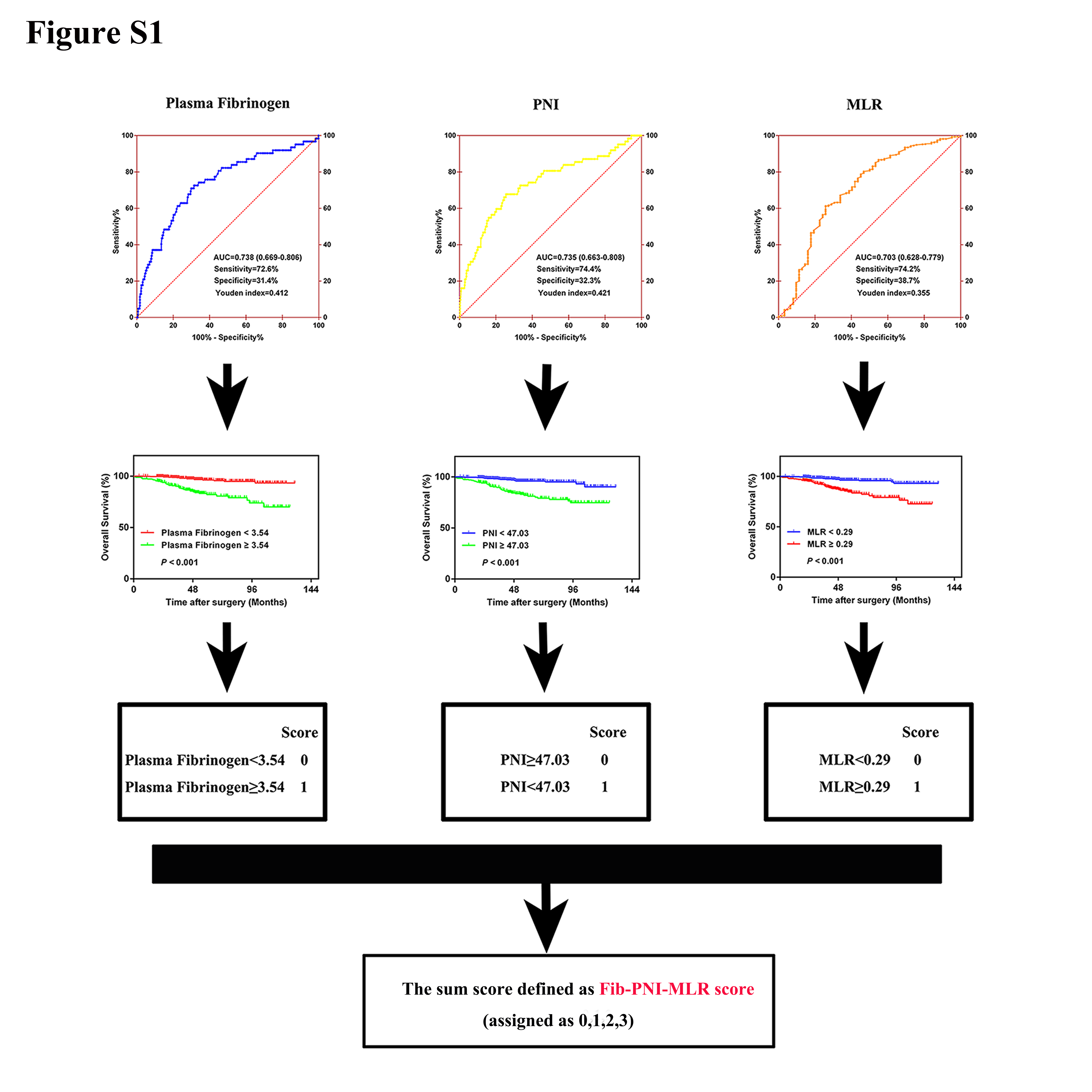

Supplement: Supplementary Figure 1 — The Fib-PNI-MLR score system was developed based on fibrinogen, PNI, and MLR. The optimal cutoff values of individual component were determined by performing receiver operating characteristic (ROC) curve analysis using the Youden index. Kaplan-Meier curves showed patients’ overall survival (OS) with higher score (score 1) indicating poorer prognosis. The Fib-PNI-MLR score was determined as the sum score of 0 or 1 according to each cutoff value. [file Image_1.tif]

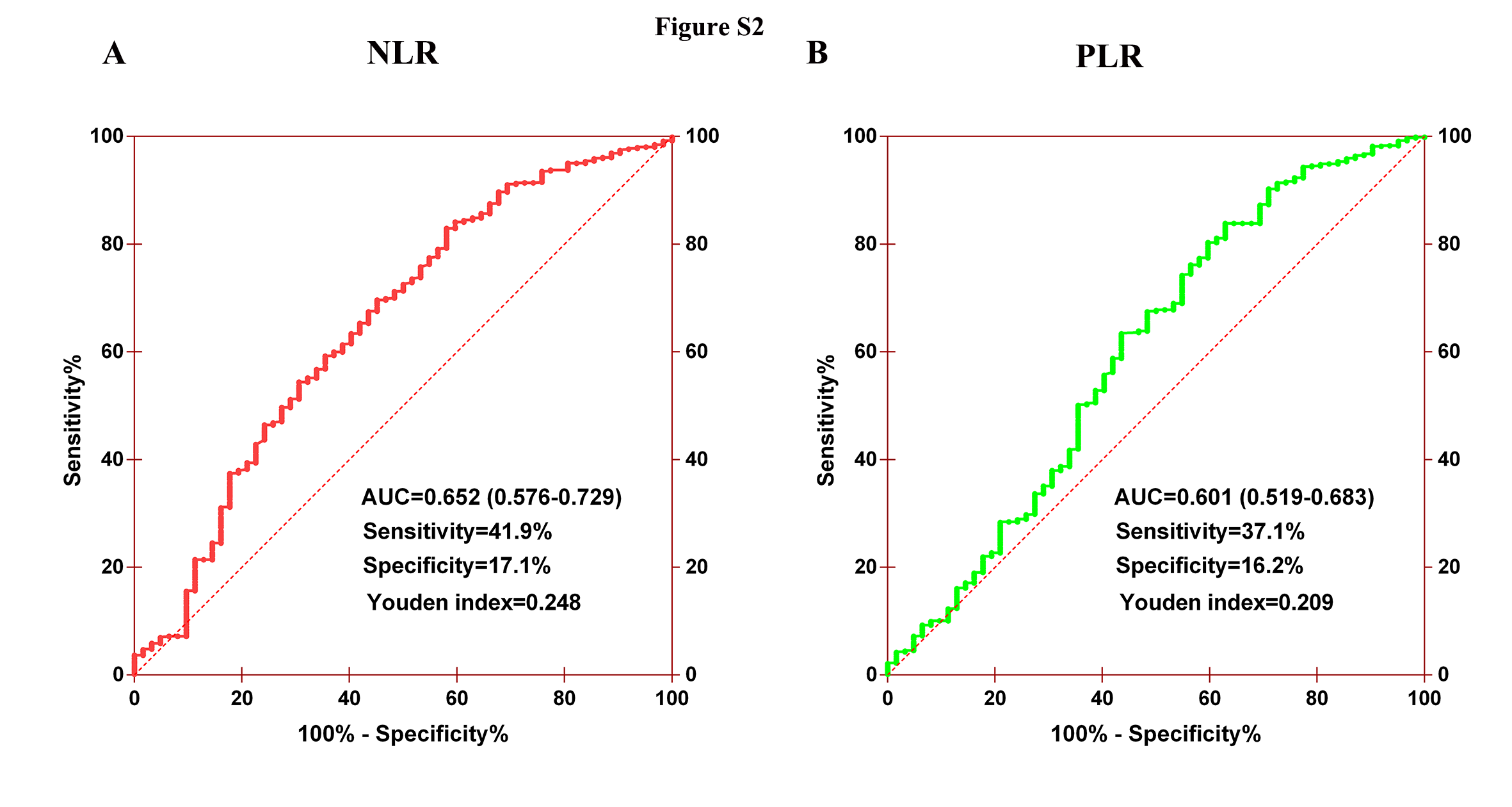

Supplement: Supplementary Figure 2 — ROC curves for NLR (A) and PLR (B). The optimal cutoff values of NLR and PLR were determined by performing receiver operating characteristic (ROC) curve analysis using the Youden index. [file Image_2.tif]

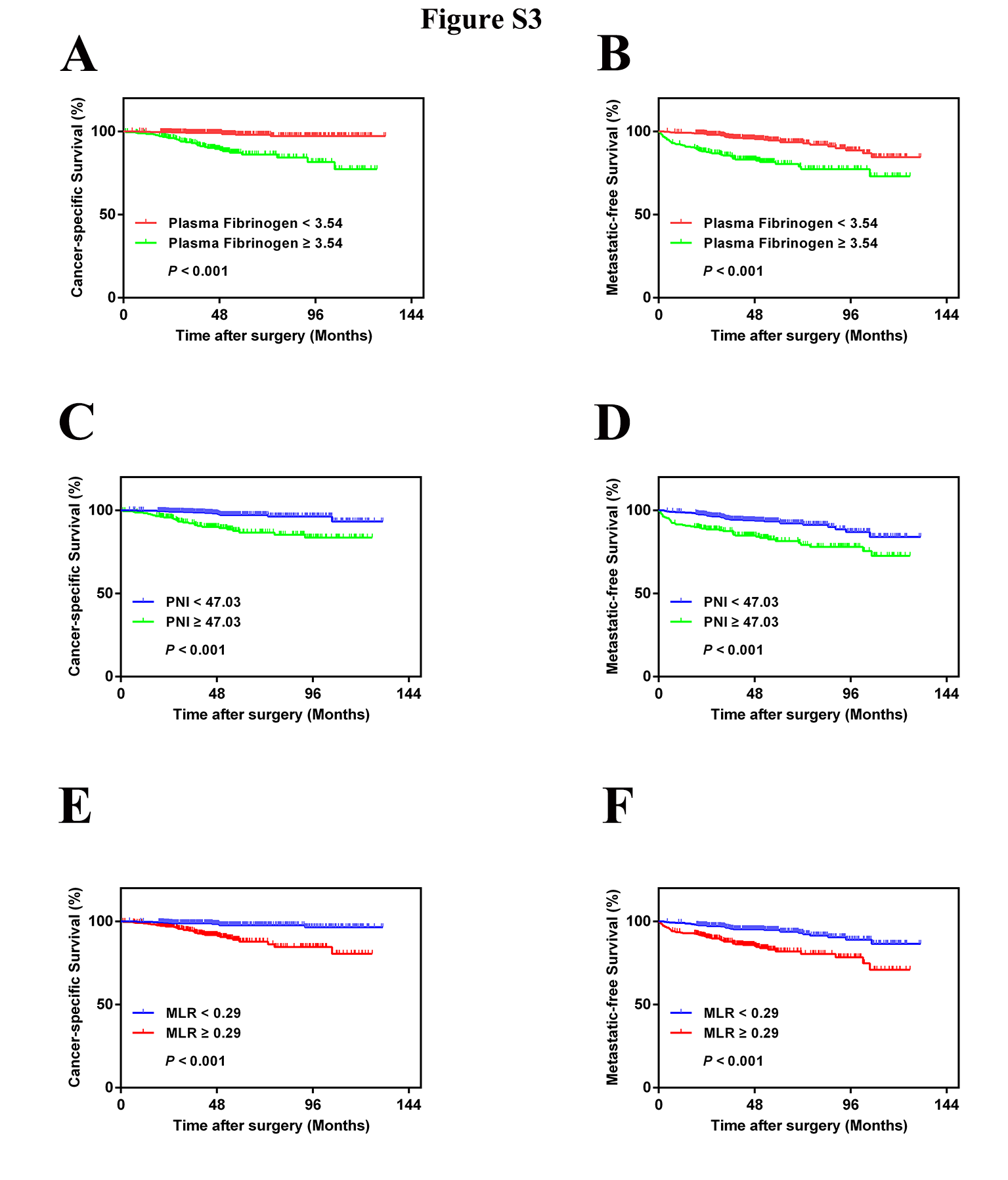

Supplement: Supplementary Figure 3 — Kaplan-Meier analysis of CSS and MFS in patients stratified by fibrinogen, MLR, and PLR. [file Image_3.tif]

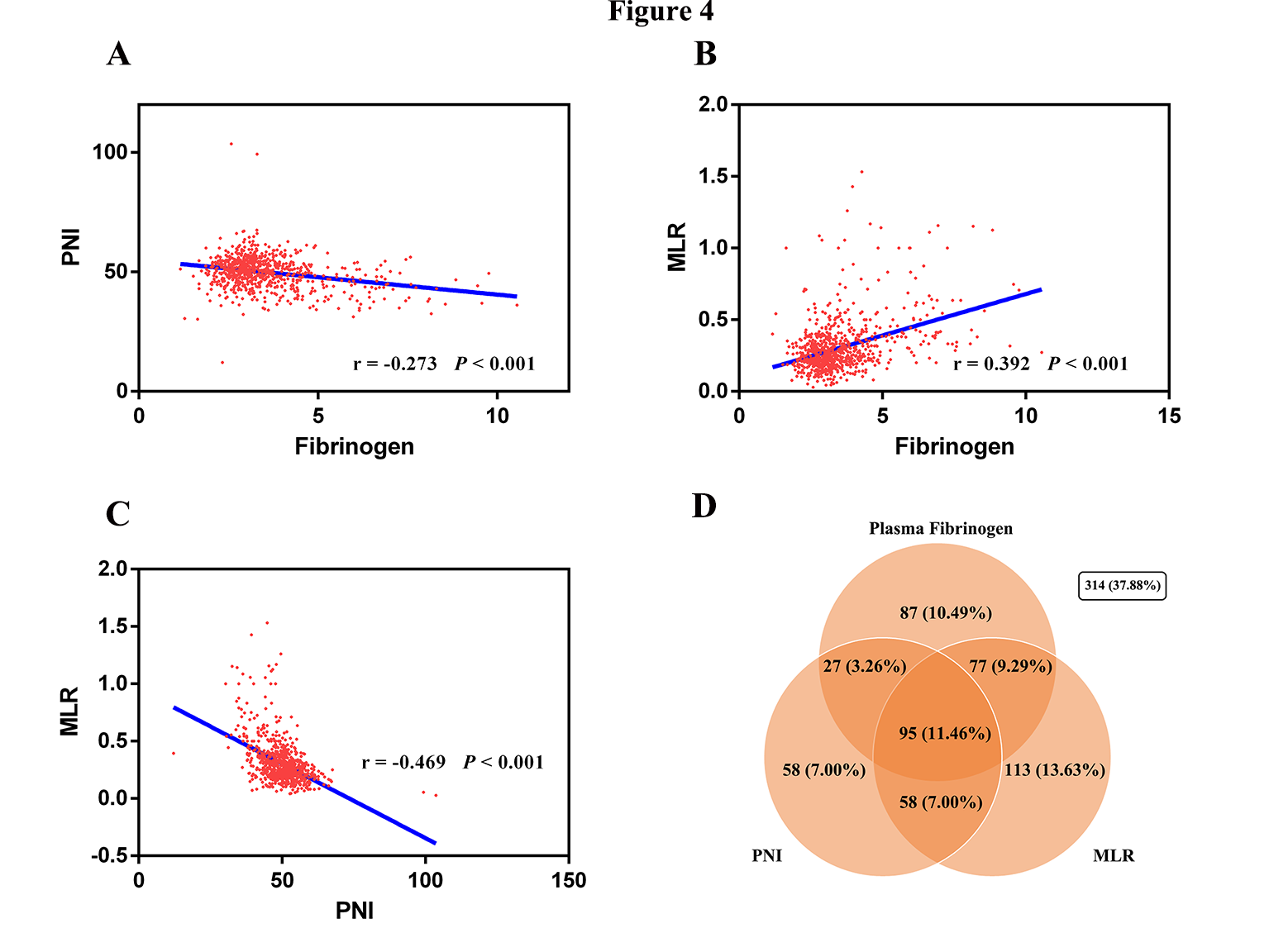

Supplement: Supplementary Figure 4 — Significant correlation between fibrinogen and PNI (A) (r = −0.273, P < 0.001), fibrinogen and MLR (B) (r = 0.392, P < 0.001), PNI and MLR (C) (r = −0.469, P < 0.001). In addition, a venn diagram of each population according to the Fib-PNI-MLR score was shown (D). [file Image_4.tif]

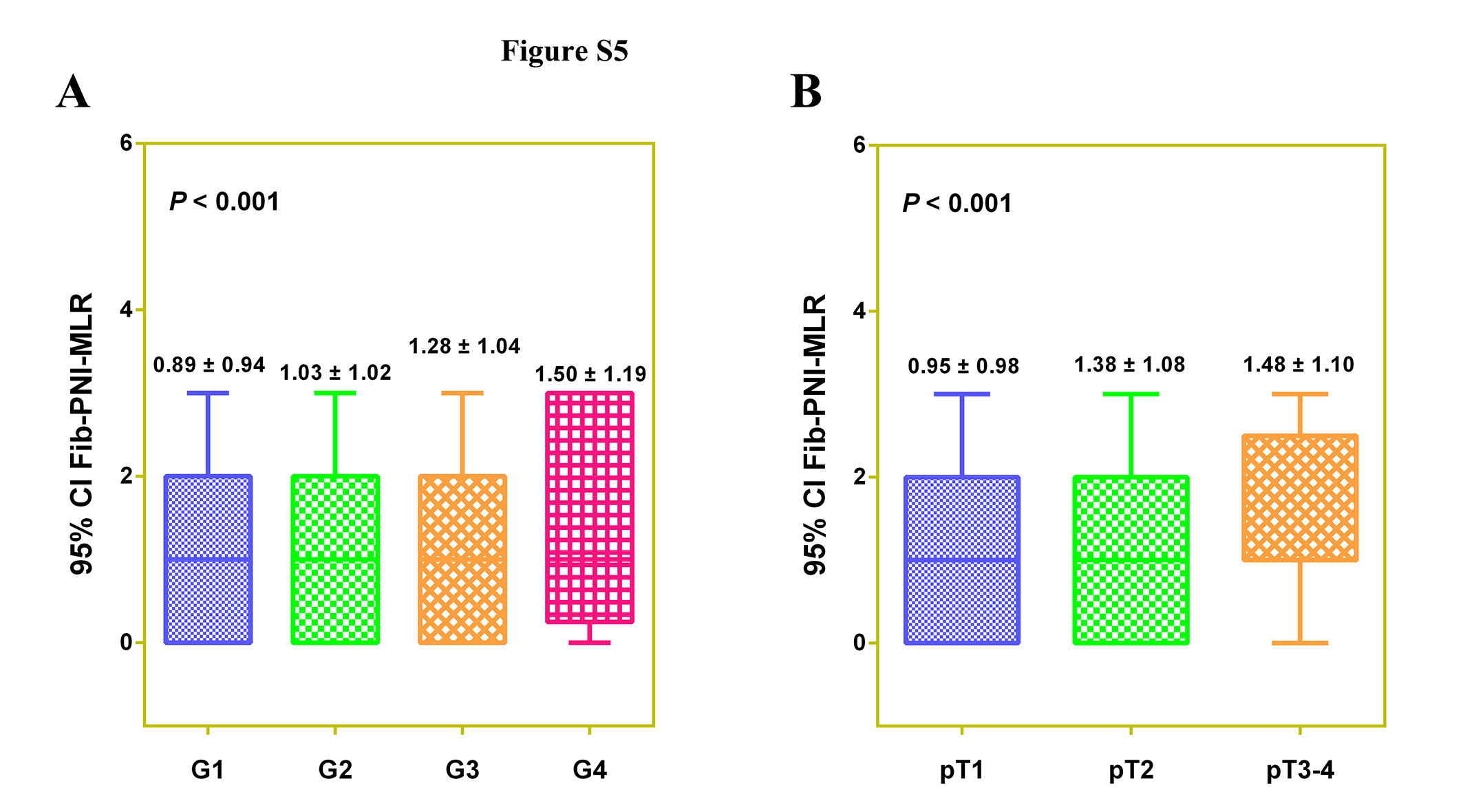

Supplement: Supplementary Figure 5 — The distribution of Fib-PNI-MLR score according to tumor grade (A) and pathologic T stage (B), respectively. [file Image_5.tif]

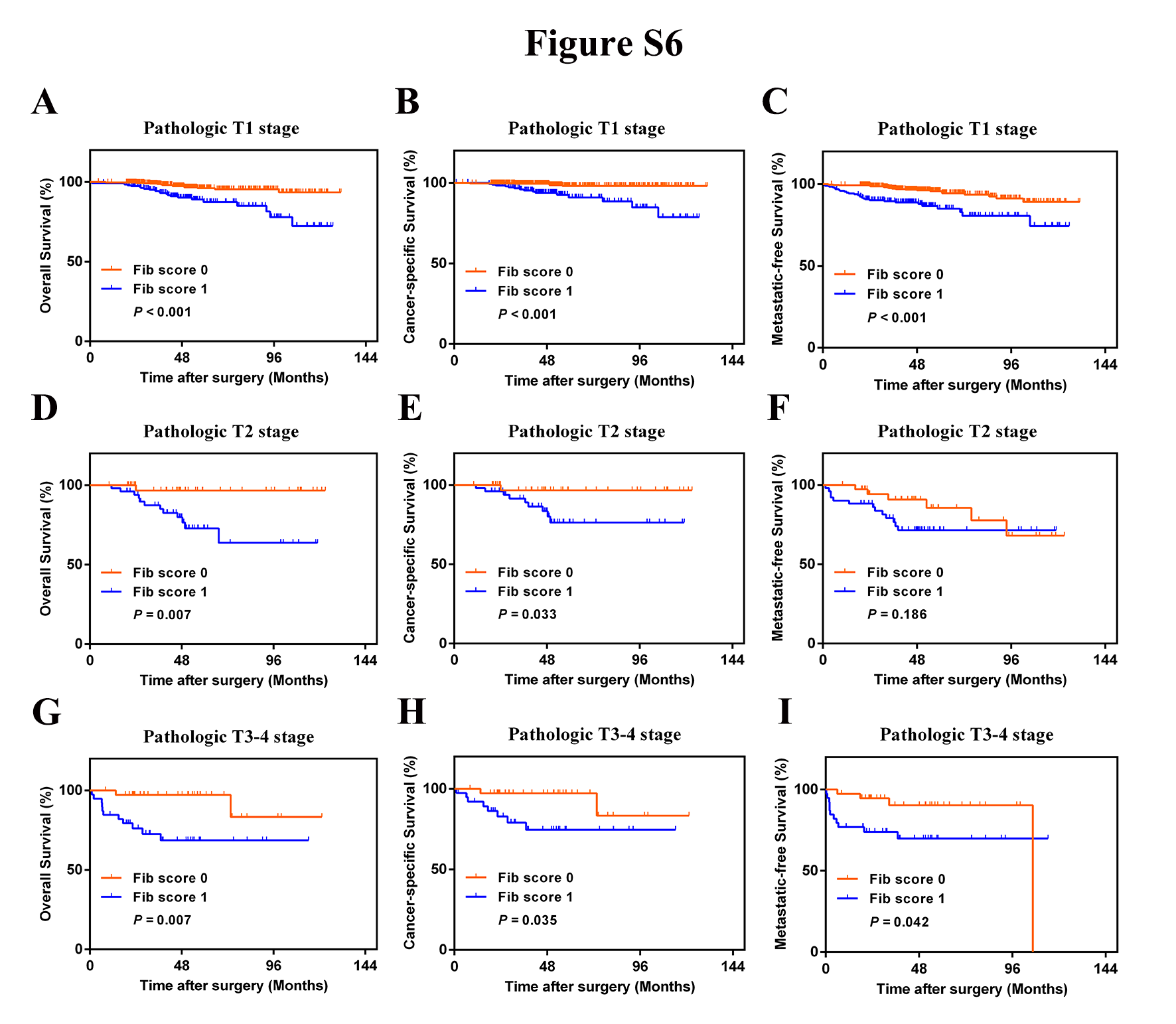

Supplement: Supplementary Figure 6 — Kaplan-Meier analysis shows that the OS, CSS, and MFS of patients with fibrinogen scores of 1 were lower than those with fibrinogen scores of 0 under adjusted pathologic T stage (T1, T2, and T3–4). [file Image_6.tif]

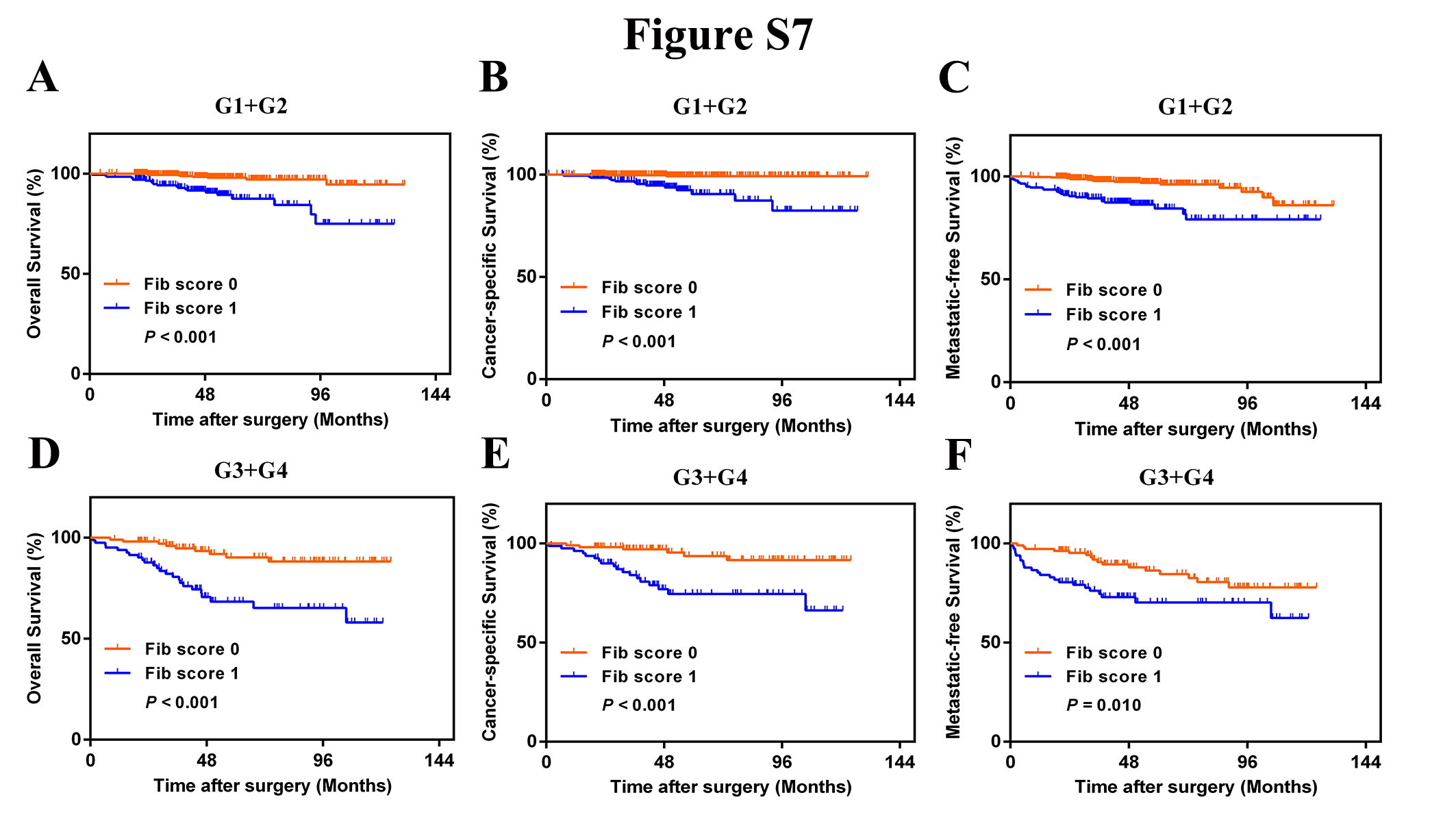

Supplement: Supplementary Figure 7 — Kaplan-Meier analysis shows that the OS, CSS, and MFS of patients with fibrinogen scores of 1 were lower than those with fibrinogen scores of 0 under tumor grade (G1+G2 and G3+G4). [file Image_7.tif]

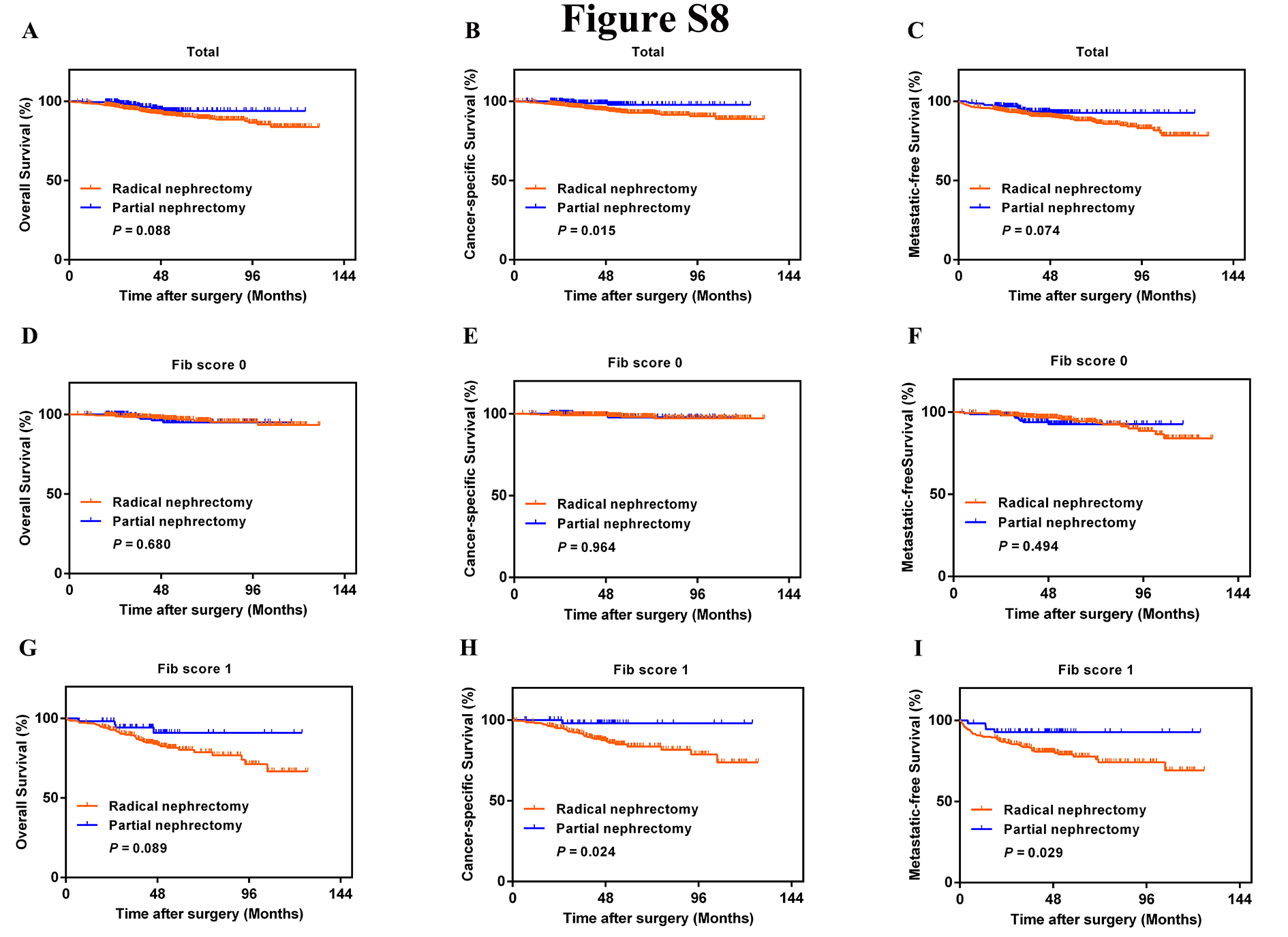

Supplement: Supplementary Figure 8 — Kaplan-Meier analysis of OS, CSS, and MFS according to the treatment options in patients with all of the fibrinogen (A–C), the fibrinogen score 0 (D–F), and the fibrinogen score 1 (G–I), respectively. [file Image_8.tif]

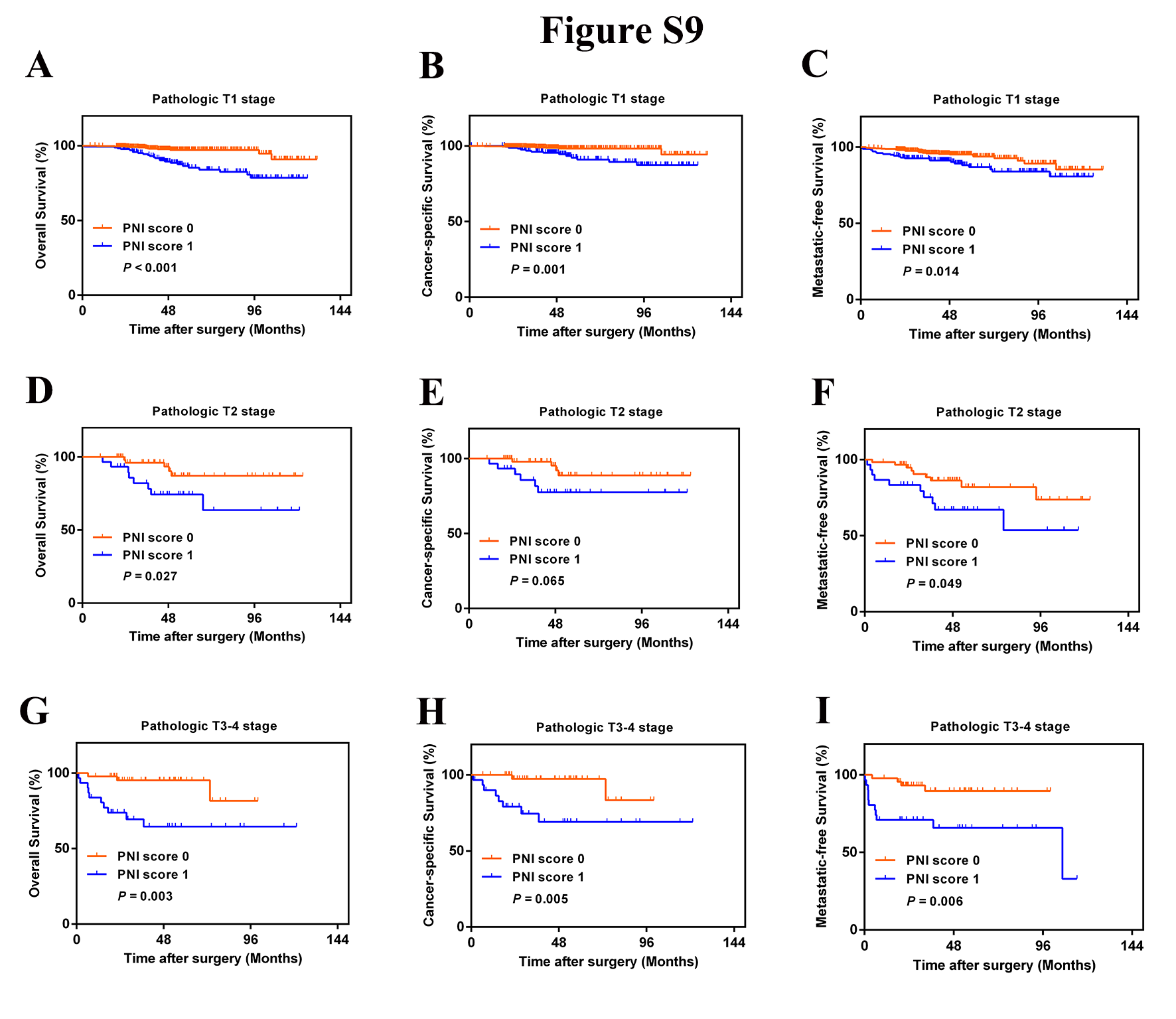

Supplement: Supplementary Figure 9 — Kaplan-Meier analysis shows that the OS, CSS, and MFS of patients with PNI scores of 1 were lower than those with PNI scores of 0 under adjusted pathologic T stage (T1, T2, and T3–4). [file Image_9.tif]

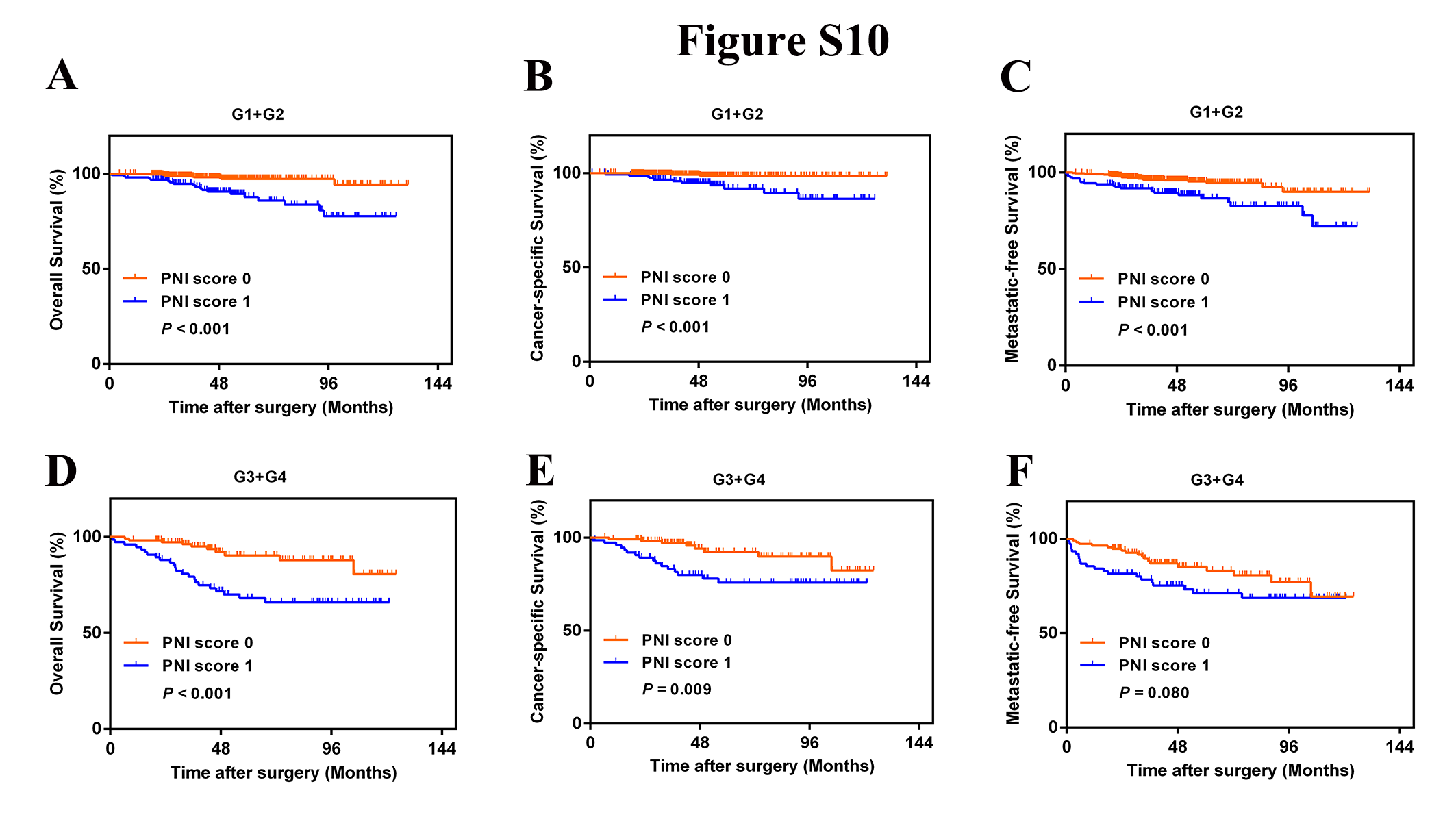

Supplement: Supplementary Figure 10 — Kaplan-Meier analysis shows that the OS, CSS, and MFS of patients with PNI scores of 1 were lower than those with PNI scores of 0 under tumor grade (G1+G2 and G3+G4). [file Image_10.tif]

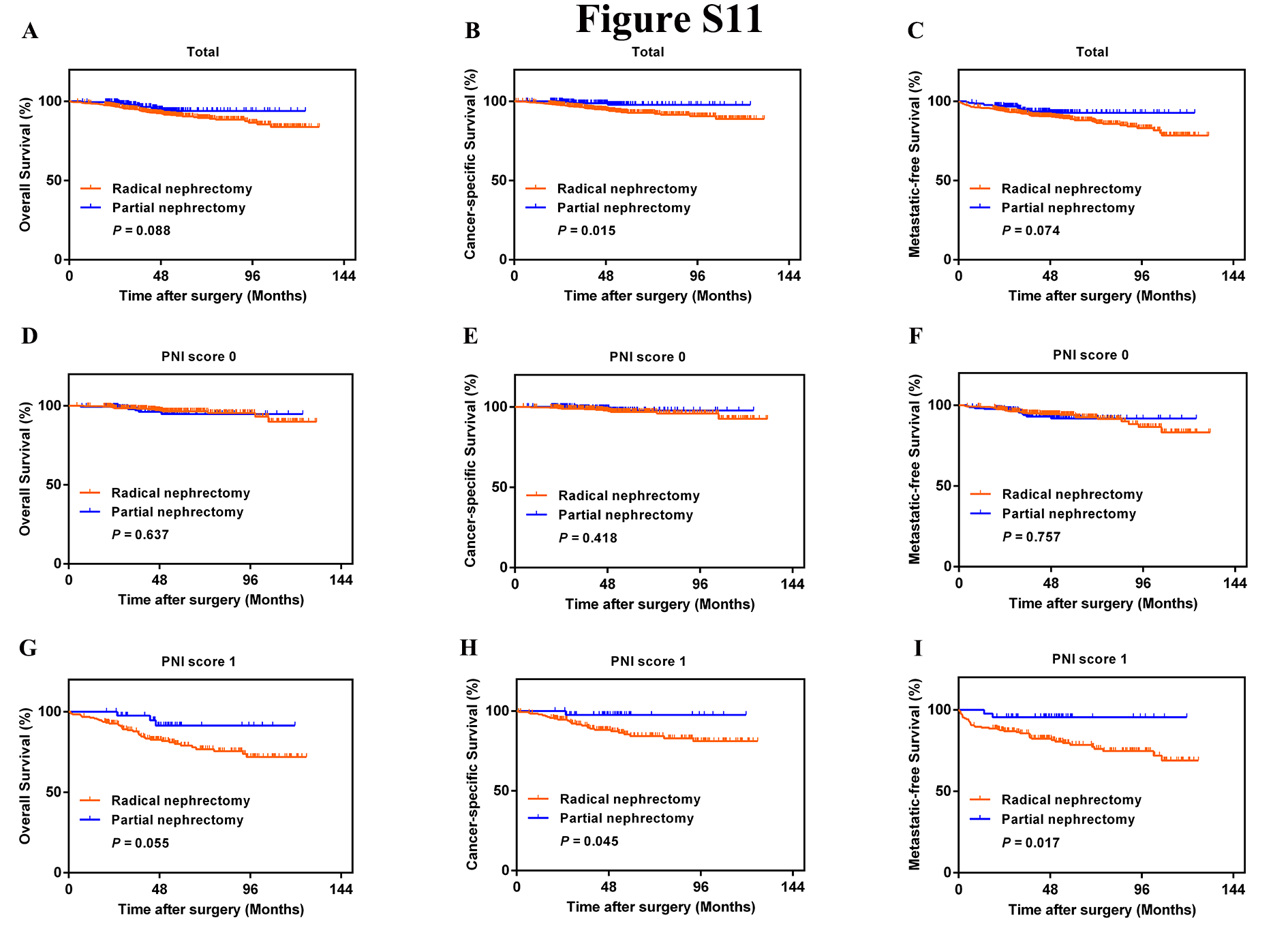

Supplement: Supplementary Figure 11 — Kaplan-Meier analysis of OS, CSS, and MFS according to the treatment options in patients with all of the PNI (A–C), the PNI score 0 (D–F), and the PNI score 1 (G–I), respectively. [file Image_11.tif]

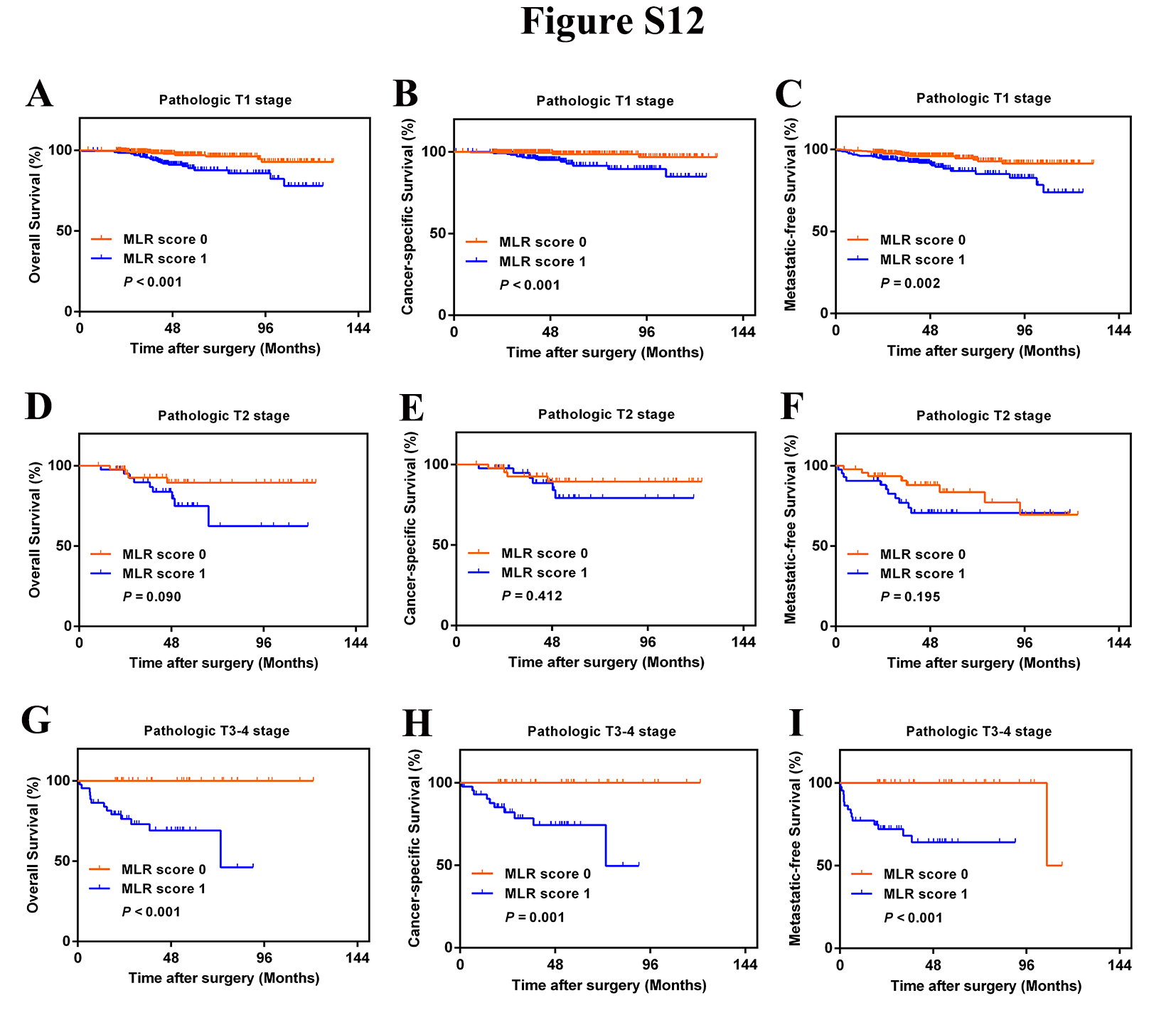

Supplement: Supplementary Figure 12 — Kaplan-Meier analysis shows that the OS, CSS, and MFS of patients with MLR scores of 1 were lower than those with MLR scores of 0 under adjusted pathologic T stage (T1, T2, and T3–4). [file Image_12.tif]

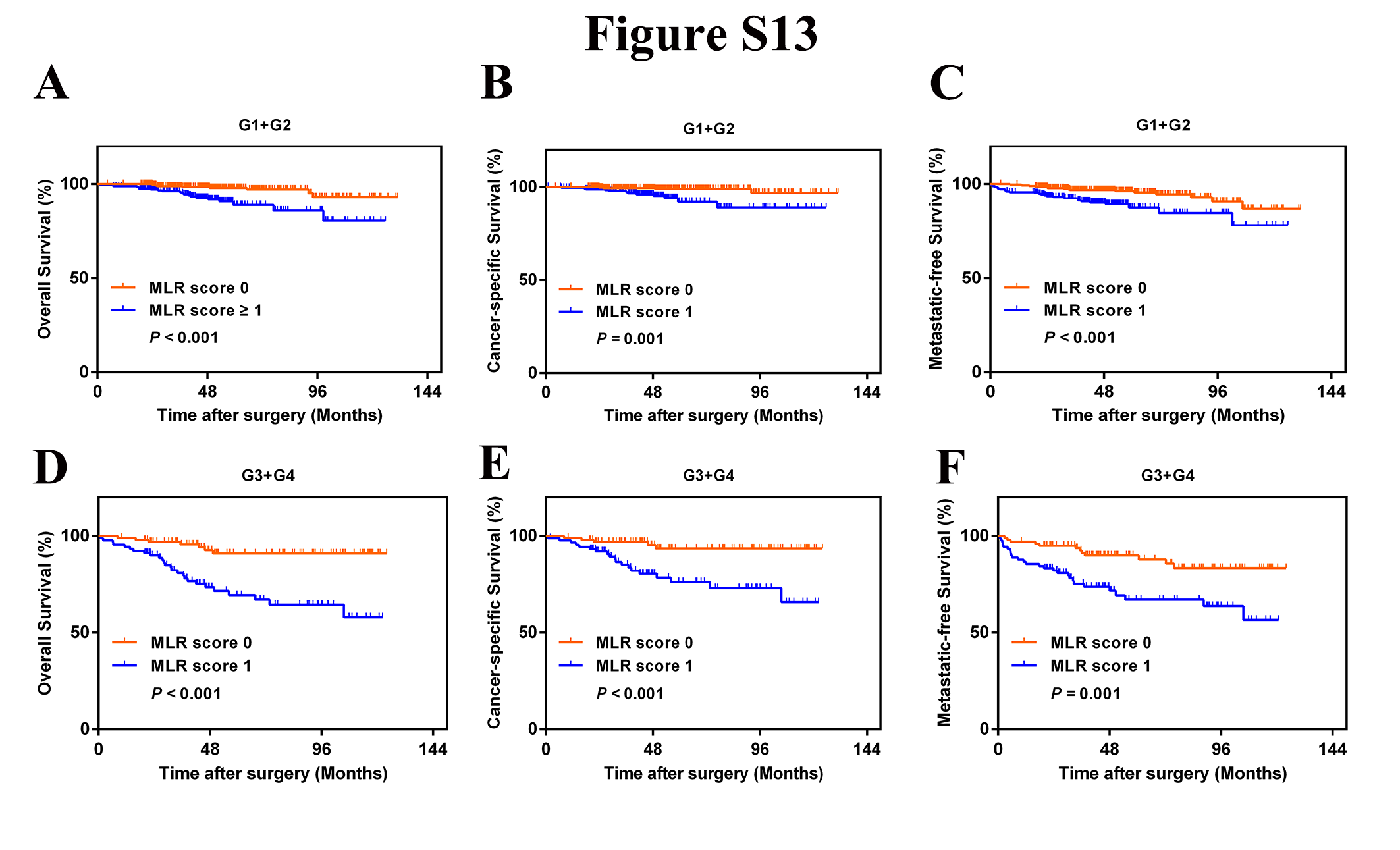

Supplement: Supplementary Figure 13 — Kaplan-Meier analysis shows that the OS, CSS, and MFS of patients with MLR scores of 1 were lower than those with MLR scores of 0 under tumor grade (G1+G2 and G3+G4). [file Image_13.tif]

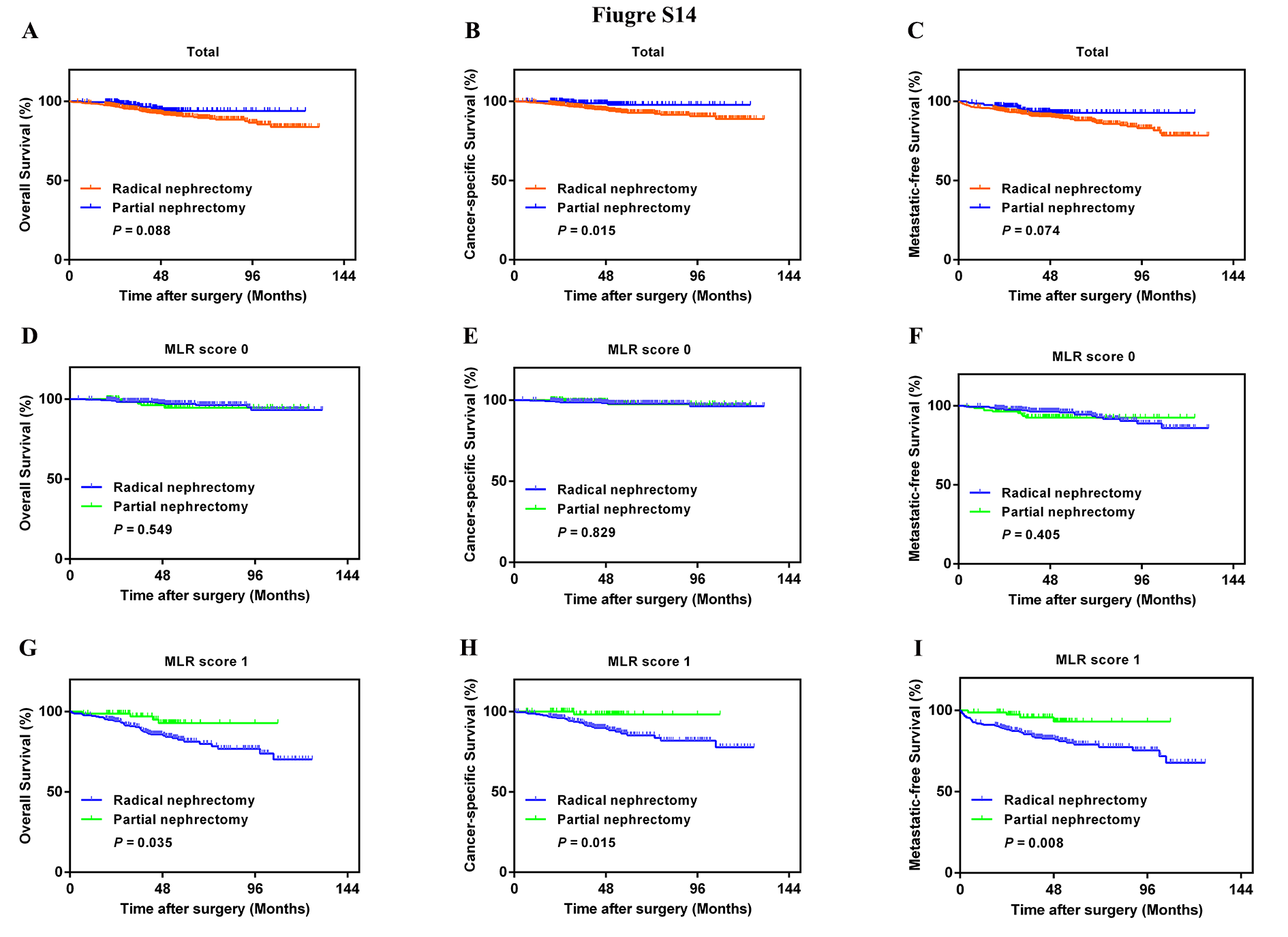

Supplement: Supplementary Figure 14 — Kaplan-Meier analysis of OS, CSS, and MFS according to the treatment options in patients with all of the MLR (A–C), the MLR score 0 (D–F), and the MLR score 1 (G–I), respectively. [file Image_14.tif]
