# Supplementary material for: The Fib-PNI-MLR Score, an Integrative Model of Coagulation Cascades, Nutrition Status, and Systemic Inflammatory Response, Predicts Urological Outcomes After Surgery in Patients With Non-Metastatic Renal Cell Carcinoma
Source: Front Oncol. 2021 Jan 5;10:555152. doi: 10.3389/fonc.2020.555152 (PMC7819501; doi:10.3389/fonc.2020.555152)
Supplement: Supplementary file 15 [file Table_1.docx]

Table S1 Clinicopathological data of patients with non-metastatic RCC

| Parameter | Total (n = 829) |
| --- | --- |
| Age, years (≥65 /<65) | 352/477 |
| Gender (male/female) | 526/303 |
| ASA grade (1/ 2/ 3) | 107/666/56 |
| BMI, kg/m^2^ (≥25/<25) | 222/607 |
| DM (yes/no) | 320/508 |
| Hypertension (yes/no) | 355/474 |
| Anemia (yes/no) | 104/725 |
| Hypoalbuminemia (yes/no) | 96/733 |
| Surgical approach (Partial nephrectomy/ Radical nephrectomy) | 221/608 |
| CKD stage (CKD1/CKD2/CKD3/CKD4/CKD5) | 567/219/28/3/12 |
| Pathologic T stage (pT1/pT2/pT3/pT4) | 662/90/68/9 |
| Fuhrman grade (1/2/3/4) | 281/358/170/20 |
| Histologic subtype (Clear cell/ Papillary/ Chromophobe/ Collecting duct/ Unclassified) | 714/57/54/1/3 |
| Tumor necrosis (yes/no) | 32/797 |
| Tumor size, cm (≥7/<7) | 141/688 |

RCC: renal cell carcinoma; DM, diabetes mellitus; CKD, chronic kidney disease.
