# Supplementary material for: The Fib-PNI-MLR Score, an Integrative Model of Coagulation Cascades, Nutrition Status, and Systemic Inflammatory Response, Predicts Urological Outcomes After Surgery in Patients With Non-Metastatic Renal Cell Carcinoma
Source: Front Oncol. 2021 Jan 5;10:555152. doi: 10.3389/fonc.2020.555152 (PMC7819501; doi:10.3389/fonc.2020.555152)
Supplement: Supplementary file 16 [file Table_2.docx]

Table S2 Clinical laboratory data and survival outcomes in 829 patients with non-metastatic RCC

| Parameter | Total (n = 829) |
| --- | --- |
| Age, years | 60.37 ± 12.45 |
| BMI, kg/m^2^ | 23.32 ± 3.18 |
| Tumor size, cm | 4.79 ± 3.20 |
| Serum creatinine, mg/dl | 0.92 ± 1.10 |
| BUN, mg/dl | 5.87 ± 2.72 |
| Uric acid, mg/dl | 5.63 ± 1.52 |
| ALT, U/l | 25.77 ± 25.19 |
| AST, U/l | 25.87 ± 15.28 |
| ALP, U/l | 80.14 ± 35.51 |
| Hemoglobin, g/dl | 133.55 ± 18.20 |
| Albumin, g/l | 41.00 ± 4.99 |
| Plasma fibrinogen, mg/dl | 3.51 ± 1.25 |
| WBCs, cells × 10^3^/ul | 6.56 ± 2.06 |
| Neutrophils, cells/ul | 4.10 ± 1.69 |
| Monocytes, cells/ul | 0.49 ± 0.20 |
| Lymphocytes, cells/ul | 1.80 ± 0.78 |
| Platelet, cells × 10^4^/ul | 220.89 ± 70.78 |
| PNI | 50.40 ± 6.67 |
| NLR | 2.20 ± 1.96 |
| MLR | 0.26 ± 0.18 |
| PLR | 123.68 ± 61.44 |
| All-cause death, n (%) | 62 (7.5%) |
| Follow up duration, months, median (quartile) | 48.8 (32.65-69.30) |
| 1 year OS rate | 98.9% |
| 3 year OS rate | 95.6% |
| 5 year OS rate | 91.3% |
| 10 year OS rate | 85.4% |
| Cancer-specific death, n (%) | 40 (4.8%) |
| Follow up duration, months, median (quartile) | 48.8 (32.65-69.30) |
| 1 year CSS rate | 99.4% |
| 3 year CSS rate | 97.1% |
| 5 year CSS rate | 93.9% |
| 10 year CSS rate | 90.5% |
| Patients who developed metastasis after surgery, n (%) | 82 (9.9%) |
| Follow up duration, months, median (quartile) | 47.5 (31.05-67.15) |
| 1 year MFS rate | 96.6% |
| 3 year MFS rate | 92.5% |
| 5 year MFS rate | 89.8% |
| 10 year MFS rate | 80.5% |

RCC, renal cell carcinoma; OS, overall survival; CSS, cancer-specific survival; MFS, metastatic-free survival; WBC, white blood cell; PNI, prognostic nutritional index; NLR, neutrophil-lymphocyte ratio; PLR, platelet-lymphocyte ratio; MLR, monocyte-lymphocyte ratio; ALT, alanine aminotransferase; AST, glutamate aminotransferase; ALP, alkaline phosphatase; BUN, urea nitrogen.
