# Supplementary material for: The Fib-PNI-MLR Score, an Integrative Model of Coagulation Cascades, Nutrition Status, and Systemic Inflammatory Response, Predicts Urological Outcomes After Surgery in Patients With Non-Metastatic Renal Cell Carcinoma
Source: Front Oncol. 2021 Jan 5;10:555152. doi: 10.3389/fonc.2020.555152 (PMC7819501; doi:10.3389/fonc.2020.555152)
Supplement: Supplementary file 17 [file Table_3.docx]

Table S3 Univariate analysis of parameters for the prediction of survival outcomes in 829 non-metastatic RCC patients

| Parameter | Overall survival | | | Cancer-specific survival | | | Metastasis-free survival | | |
| --- | --- | --- | --- | --- | --- | --- | --- | --- | --- |
|  | HR | 95%CI | *P* value | HR | 95%CI | *P* value | HR | 95%CI | *P* value |
| Age, years (≥65 /<65) | 3.099 | 1.790-5.364 | <0.001* | 2.662 | 1.373-5.159 | 0.004* | 2.044 | 1.311-3.187 | 0.002* |
| Gender (male/female) | 1.321 | 0.770-2.267 | 0.313 | 0.878 | 0.466-1.653 | 0.687 | 1.733 | 1.055-2.847 | 0.030* |
| ASA grade (≥3/<3) | 4.225 | 2.291-7.791 | <0.001* | 3.994 | 1.839-8.673 | <0.001* | 2.700 | 1.463-4.982 | 0.001* |
| BMI, kg/m^2^ (≥25/<25) | 0.359 | 0.163-0.788 | 0.011* | 0.497 | 0.209-1.185 | 0.115 | 0.382 | 0.197-0.739 | 0.004* |
| DM (yes/no) | 0.938 | 0.556-1.581 | 0.810 | 0.733 | 0.372-1.445 | 0.370 | 0.744 | 0.464-1.193 | 0.220 |
| Hypertension (yes/no) | 1.199 | 0.726-1.981 | 0.478 | 1.322 | 0.709-2.463 | 0.380 | 1.390 | 0.900-2.147 | 0.138 |
| Anemia (yes/no) | 5.066 | 3.058-8.393 | <0.001* | 3.381 | 1.743-6.556 | <0.001* | 2.995 | 1.860-4.823 | <0.001* |
| Hypoalbuminemia (yes/no) | 4.151 | 2.473-6.966 | <0.001* | 3.178 | 1.626-6.209 | 0.001* | 2.298 | 1.389-3.804 | 0.001* |
| Surgical approach (Partial nephrectomy/ Radical nephrectomy) | 0.544 | 0.268-1.106 | 0.093 | 0.258 | 0.079-0.839 | 0.024* | 0.585 | 0.323-1.061 | 0.078 |
| CKD stage |  |  |  |  |  |  |  |  |  |
| CKD1 | 1.000 | Reference | 1.000 | 1.000 | Reference | 1.000 | 1.000 | Reference | 1.000 |
| CKD2-3 | 4.043 | 1.734-9.426 | 0.001* | 4.086 | 1.450-11.518 | 0.008* | 2.926 | 1.270-6.741 | 0.012* |
| CKD4-5 | 6.035 | 2.176-16.733 | 0.001* | 2.239 | 0.306-16.385 | 0.427 | 6.957 | 3.014-16.054 | <0.001* |
| Pathologic T stage |  |  |  |  |  |  |  |  |  |
| pT1 | 1.000 | Reference | 1.000 | 1.000 | Reference | 1.000 | 1.000 | Reference | 1.000 |
| pT2 | 2.683 | 1.422-5.065 | 0.002* | 3.750 | 1.753-8.022 | 0.001 | 3.085 | 1.182-5.251 | <0.001* |
| pT3 | 3.497 | 1.734-7.056 | <0.001* | 4.978 | 2.189-11.318 | <0.001* | 3.081 | 1.634-5.807 | 0.001* |
| pT4 | 9.551 | 2.933-31.102 | <0.001* | 11.344 | 2.644-48.674 | 0.001* | 6.836 | 2.125-21.994 | 0.001* |
| Fuhrman grade (≥3/<3) | 3.524 | 2.133-5.824 | <0.001* | 4.692 | 2.483-8.866 | <0.001* | 2.872 | 1.856-4.444 | <0.001* |
| Histologic subtype (Clear cell/non- Clear cell) | 1.645 | 0.875-3.093 | 0.122 | 1.989 | 0.946-4.183 | 0.070 | 1.396 | 0.785-2.483 | 0.257 |
| Tumor necrosis (yes/no) | 1.856 | 0.673-5.118 | 0.232 | 2.954 | 1.050-8.308 | 0.040* | 2.259 | 0.983-5.191 | 0.055 |
| Tumor size, cm (≥7/<7) | 2.429 | 1.426-4.138 | 0.001* | 3.774 | 2.015-7.068 | <0.001* | 2.555 | 1.611-4.050 | <0.001* |
| Fib-PNI-MLR |  |  |  |  |  |  |  |  |  |
| 0-1 | 1.000 | Reference | 1.000 | 1.000 | Reference | 1.000 | 1.000 | Reference | 1.000 |
| 2 | 5.053 | 2.533-10.078 | <0.001* | 6.921 | 2.761-17.350 | <0.001* | 2.088 | 1.172-3.720 | 0.013* |
| 3 | 13.731 | 7.254-25.991 | <0.001* | 19.002 | 8.033-44.948 | <0.001* | 7.209 | 4.414-11.775 | <0.001* |
| NLR (≥3.30/<3.30) | 3.175 | 1.917-5.258 | <0.001* | 4.412 | 2.374-8.202 | <0.001* | 3.274 | 2.110-5.080 | <0.001* |
| PLR (≥184.16/<184.16) | 2.970 | 1.773-4.974 | <0.001* | 3.724 | 1.988-6.975 | <0.001* | 3.030 | 1.932-4.753 | <0.001* |
| BUN (≥7.2/<7.2), mg/dl | 1.885 | 1.053-3.372 | 0.033* | 1.473 | 0.679-3.199 | 0.327 | 2.324 | 1.435-3.765 | 0.001* |
| Hyperuricemia (yes/no) | 0.989 | 0.515-1.898 | 0.974 | 0.975 | 0.431-2.204 | 0.951 | 1.099 | 0.636-1.897 | 0.736 |
| ALT (≥40/<40), U/l | 0.381 | 0.119-1.215 | 0.103 | 0.041 | 0.001-2.758 | 0.137 | 0.381 | 0.139-1.039 | 0.059 |
| AST (≥35/<35), U/l | 0.983 | 0.508-1.904 | 0.960 | 0.508 | 0.179-1.440 | 0.202 | 0.866 | 0.474-1.581 | 0.639 |
| ALP (≥125/<125), U/l | 1.852 | 0.795-4.314 | 0.153 | 1.397 | 0.429-4.549 | 0.578 | 1.397 | 0.606-3.218 | 0.432 |

**P*<0.05

RCC: renal cell carcinoma; DM, diabetes mellitus; CKD, chronic kidney disease; OS, overall survival; CSS, cancer-specific survival; MFS, metastatic-free survival; PNI, prognostic nutritional index; NLR, neutrophil-lymphocyte ratio; PLR, platelet-lymphocyte ratio; ALT, alanine aminotransferase; AST, glutamate aminotransferase; ALP, alkaline phosphatase; BUN, urea nitrogen.
