# Supplementary material for: The Fib-PNI-MLR Score, an Integrative Model of Coagulation Cascades, Nutrition Status, and Systemic Inflammatory Response, Predicts Urological Outcomes After Surgery in Patients With Non-Metastatic Renal Cell Carcinoma
Source: Front Oncol. 2021 Jan 5;10:555152. doi: 10.3389/fonc.2020.555152 (PMC7819501; doi:10.3389/fonc.2020.555152)
Supplement: Supplementary file 18 [file Table_4.docx]

Table S4 Multivariate analysis of parameters for the prediction of survival outcomes in 829 non-metastatic RCC patients when excluding Fib-PNI-MLR

| Parameter | Overall survival | | | Cancer-specific survival | | | Metastasis-free survival | | |
| --- | --- | --- | --- | --- | --- | --- | --- | --- | --- |
|  | HR | 95%CI | *P* value | HR | 95%CI | *P* value | HR | 95%CI | *P* value |
| Age, years (≥65 /<65) | 2.183 | 1.222-3.898 | 0.008* | 1.855 | 0.918-3.747 | 0.085 | 1.542 | 0.958-2.480 | 0.074 |
| Gender (male/female) |  | - |  |  | - |  | 1.669 | 0.995-2.801 | 0.052 |
| ASA grade (≥3/<3) | 1.678 | 0.840-3.352 | 0.143 | 1.704 | 0.688-4.223 | 0.250 | 1.162 | 0.587-2.299 | 0.667 |
| BMI, kg/m^2^ (≥25/<25) | 0.545 | 0.238-1.248 | 0.151 |  | - |  | 0.413 | 0.208-0.820 | 0.011* |
| Anemia (yes/no) | 1.938 | 1.071-3.505 | 0.029* | 1.297 | 0.616-2.731 | 0.494 | 1.184 | 0.666-2.105 | 0.566 |
| Hypoalbuminemia (yes/no) | 1.481 | 0.833-2.632 | 0.181 | 1.086 | 0.508-2.318 | 0.832 | 1.021 | 0.577-1.806 | 0.943 |
| Surgical approach (Partial nephrectomy/ Radical nephrectomy) |  | - |  | 0.555 | 0.160-1.928 | 0.354 |  | - |  |
| CKD stage |  |  |  |  |  |  |  |  |  |
| CKD1 | 1.000 | Reference | 1.000 | 1.000 | Reference | 1.000 | 1.000 | Reference | 1.000 |
| CKD2-3 vs. CKD1 | 1.958 | 0.696-5.510 | 0.203 | 2.015 | 0.603-6.735 | 0.255 | 1.559 | 0.584-4.161 | 0.376 |
| CKD4-5 vs. CKD1 | 3.402 | 0.994-11.645 | 0.051 | 1.191 | 0.150-9.438 | 0.869 | 3.347 | 1.177-9.522 | 0.024* |
| Pathologic T stage |  |  |  |  |  |  |  |  |  |
| pT1 | 1.000 | Reference | 1.000 | 1.000 | Reference | 1.000 | 1.000 | Reference | 1.000 |
| pT2 vs pT1 | 1.446 | 0.590-3.544 | 0.420 | 1.300 | 0.468-3.607 | 0.614 | 1.827 | 0.841-3.968 | 0.128 |
| pT3 vs pT1 | 2.198 | 1.009-4.789 | 0.047* | 2.491 | 0.966-6.423 | 0.059 | 2.176 | 1.099-4.310 | 0.026* |
| pT4 vs pT1 | 8.632 | 2.278-32.716 | 0.002* | 8.919 | 1.787-44.521 | 0.008* | 6.684 | 1.864-23.969 | 0.004* |
| Fuhrman grade (≥3/<3) | 2.159 | 1.233-3.780 | 0.007* | 1.847 | 0.776-4.393 | 0.165 | 1.789 | 1.110-2.885 | 0.017* |
| Tumor necrosis (yes/no) |  | - |  | 1.122 | 0.339-3.718 | 0.851 |  | - |  |
| Tumor size, cm (≥7/<7) | 1.237 | 0.579-2.640 | 0.583 | 1.847 | 0.776-4.393 | 0.165 | 1.158 | 0.596-2.249 | 0.666 |
| NLR (≥3.30/<3.30) | 1.764 | 1.072-3.203 | 0.042* | 2.293 | 1.093-4.810 | 0.028* | 1.861 | 1.098-3.155 | 0.021* |
| PLR (≥184.16/<184.16) | 1.149 | 0.608-2.169 | 0.669 | 1.474 | 0.688-3.157 | 0.318 | 1.524 | 0.877-2.649 | 0.135 |
| BUN (≥7.2/<7.2), mg/dl | 0.823 | 0.381-1.779 | 0.620 |  | - |  | 1.301 | 0.706-2.399 | 0.399 |

**P*<0.05

RCC: renal cell carcinoma; DM, diabetes mellitus; CKD, chronic kidney disease; OS, overall survival; CSS, cancer-specific survival; MFS, metastatic-free survival; PNI, prognostic nutritional index; NLR, neutrophil-lymphocyte ratio; PLR, platelet-lymphocyte ratio; BUN, urea nitrogen.
